# Intrinsic Fermi Level Pinning and Reversible Depinning at Ultralow-Defect Epitaxial MoSe_2_/Au(111) Interfaces

**DOI:** 10.1021/acs.nanolett.6c02384

**Published:** 2026-09-20

**Authors:** Guan-Hao Chen, Cheng-Rong Hsing, Li-Syuan Lu, Po-Sen Mao, Pei-Yu Chuang, Yi Chou, Yu-Shiuan Su, Chih-Pin Lin, Tuo-Hung Hou, Juhn-Jong Lin, Yi-Chia Chou, Cheng-Maw Cheng, Ching-Ming Wei, Chun-Liang Lin, Wen-Hao Chang

**Affiliations:** ‡ Department of Electrophysics, 34914 National Yang Ming Chiao Tung University, Hsinchu 30010, Taiwan; § Research Center for Applied Sciences, 38017 Academia Sinica, Taipei 11529, Taiwan; ⊥ Division of Natural Science, Center for General Education, Chang Gung University, Taoyuan City 33302, Taiwan; ∥ Department of Artificial Intelligence, Chang Gung University, Taoyuan City 33302, Taiwan; ¶ Research Center for Critical Issues, Academia Sinica, Tainan 711010, Taiwan; # 57815 National Synchrotron Radiation Research Center, Hsinchu 300, Taiwan; $ Taiwan Instrument Research Institute, Hsinchu 300092, Taiwan; ∇ Department of Electronics Engineering and Institute of Electronics, National Yang Ming Chiao Tung University, Hsinchu 30010, Taiwan; ○ Department of Materials Science and Engineering, 33561 National Taiwan University, Taipei 10617, Taiwan; @ Department of Physics, National Sun Yat-sen University, Kaohsiung 80424, Taiwan; ◇ Institute of Atomic and Molecular Sciences, Academia Sinica, Taipei 106923, Taiwan; ◆ Center for Emergent Functional Matter Science (CEFMS), National Yang Ming Chiao Tung University, Hsinchu 30010, Taiwan

**Keywords:** Two-dimensional semiconductors, Transition-metal dichalcogenide, Molybdenum diselenide, Scanning tunneling microscopy, Fermi level pinning

## Abstract

Fermi level pinning (FLP) fundamentally limits contact engineering in two-dimensional semiconductors, yet its intrinsic origin remains obscured by the presence of interfacial disorder and defect-induced gap states. Although theoretical models attribute FLP to metal-induced gap states (MIGSs) and interface dipoles, direct experimental validation at an ideal van der Waals interface has remained elusive. Here, we establish an ultralow-defect epitaxial monolayer MoSe_2_/Au­(111) interface (defect density ∼ 1.7 × 10^10^ cm^–2^) to directly probe its electronic structure using scanning tunneling spectroscopy and field-emission resonance. Despite the nearly pristine interface, we observe symmetric electron and hole Schottky barriers alongside pronounced work function lowering, demonstrating that FLP persists even without significant interfacial disorder. These measurements quantitatively support the MIGS-induced interface dipole model. Remarkably, selenium intercalation reversibly suppresses the interfacial dipole, restores the work function, and shifts the Fermi level, resulting in substantial depinning and establishing a strategy for band alignment engineering at van der Waals interfaces.

## Graphical abstract

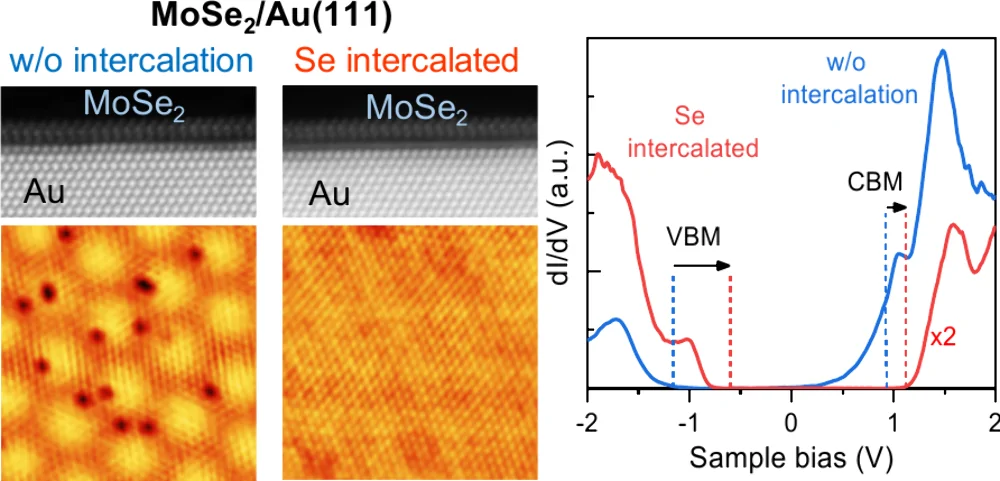

Understanding and controlling the electronic properties at two-dimensional (2D) semiconductor–metal interfaces remains a central challenge for advancing atomically thin electronics.^1–9^ A primary limitation is Fermi level pinning (FLP), which renders Schottky barrier heights (SBHs) largely insensitive to the choice of contact metal.^10–19^ While classical Schottky–Mott theory predicts barrier alignment governed by a metal work function, transition-metal dichalcogenides (TMDs) frequently exhibit strong pinning near a fixed position within the band gap.^20–23^

Density functional theory (DFT) calculations have attributed intrinsic FLP at TMD–metal interfaces to metal-induced gap states (MIGSs) and interfacial charge redistribution that generate interface dipoles,^10,24–29^ a mechanism distinct from the pinning effect at conventional metal–semiconductor interfaces.^30–32^ However, direct experimental verification of such intrinsic interfacial effects has remained elusive. Most experimental studies rely on metal deposition processes that introduce defects, interface reactions, and defect-induced gap states (DIGSs),^21,22,33–40^ complicating the interpretation of spectroscopic measurements. Moreover, recent reports of nearly ideal Schottky–Mott behavior achieved by metal transfer^41,42^ or by defect-minimized deposition schemes^15,43,44^ have suggested that FLP may arise primarily from extrinsic disorders. It therefore remains unclear whether FLP is an intrinsic property of ideal 2D–metal interfaces or a consequence of extrinsic disorder introduced during contact formation.

Here we address this question by establishing a model 2D–metal interface consisting of ultralow-defect monolayer MoSe_2_ epitaxially grown on Au(111). Using oxygen-free chemical vapor deposition (CVD) with controlled selenium (Se) supply, we suppress the formation of Se vacancies and achieve an ultralow defect density ∼ 1.7 × 10^10^ cm^–2^, as confirmed by scanning tunneling microscopy (STM). This pristine interface minimizes DIGSs and enables direct examination of the intrinsic electronic coupling between a 2D semiconductor and a metal contact.

Spectroscopic measurements using scanning tunneling spectroscopy (STS), field-emission resonance (FER), and angle-resolved photoemission spectroscopy (ARPES) reveal that the Fermi level in as-grown MoSe_2_/Au­(111) is pinned near midgap, with nearly symmetric SBHs for electrons and holes. FER further shows a pronounced reduction in the work function upon MoSe_2_ deposition, consistent with MIGSs and interface dipole formation.^25^ Remarkably, Se intercalation at the van der Waals interface reverses this behavior: The Fermi level shifts toward the valence band (VB), the work function increases by ∼0.5 eV, and the pinning is substantially suppressed. This transition is fully reversible through thermal annealing in ultrahigh vacuum (UHV), enabling a tunable band alignment via controlled intercalation. First-principles calculations corroborate that intercalation modulates the interface dipole strength and weakens MIGSs. Our results provide direct experimental evidence for intrinsic FLP in a model 2D–metal system and establish reversible interface dipole modulation as a route for band alignment engineering.

We first examine MoSe_2_ monolayers grown on the Au(111) surface by conventional CVD using MoO_2_ and Se precursors (Figure 1a). To enable epitaxial growth, a single-crystal Au(111) surface was prepared on *c*-plane sapphire substrates (see Methods; different annealing conditions are shown in Figures S1 and S2), as is evident from the electron backscatter diffraction (EBSD) analysis (Figure 1b). The large-area STM images reveal atomically flat Au terraces with well-defined step edges (Figure 1c), providing a template for MoSe_2_ epitaxial growth.

**Figure 1:**
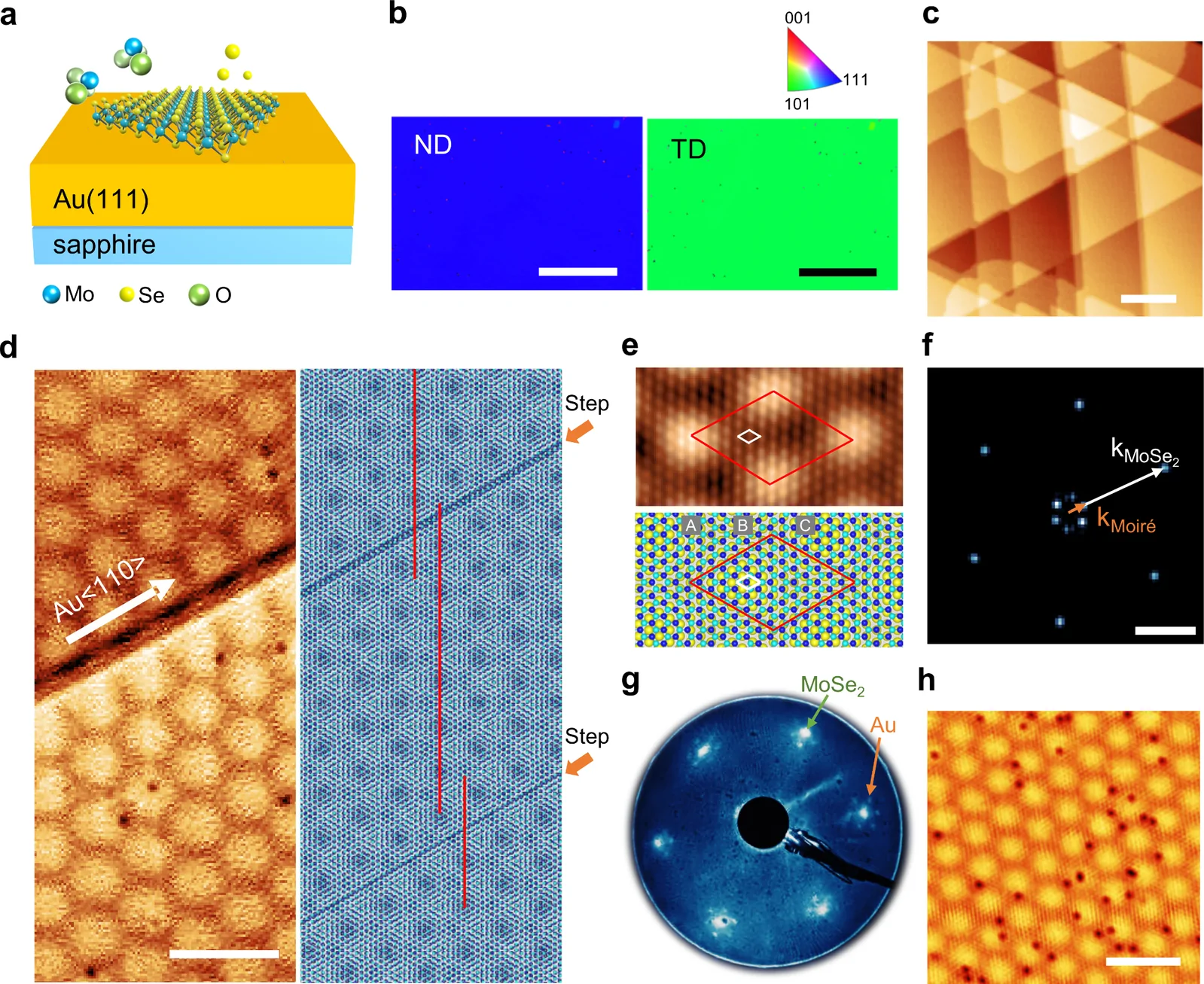
Epitaxial growth of MoSe_2_ on the Au(111) surface. (a) Schematic of the growth monolayer MoSe_2_ on Au(111) by conventional CVD growth. (b) EBSD inverse pole figure maps of single crystal Au(111) in the normal direction (ND) and transverse direction (TD). The scale bar is 250 μm. (c) Large-area STM image of a MoSe_2_ film on the Au(111) surface. The scan bias is −1 V. (d) High-resolution STM images of MoS_2_/Au­(111) with a moiré pattern across Au ⟨110⟩ step edges (left) and a ball-and-stick model for illustrating the moiré pattern across surface step edges (right). The scale bar is 4 nm. The scan bias is 0.2 V. (e) Atomically resolved STM image of moiré supercells (top) and schematic of the moiré supercell of 7 × 7 MoSe_2_ on 8 × 8 Au(111) lattices (bottom). The red and white rhombuses correspond to the moiré supercell and the unit cell of the MoSe_2_ lattice. The scan bias is −5 mV. (f) FFT analysis of the STM image shown in part e. The scale bar is 2 nm^–1^. (g) LEED pattern of the MoSe_2_ film on Au(111). (h) STM image of MoSe_2_ on Au(111) with point defects (dark spots) grown by conventional CVD. The scale bar is 4 nm. The scan bias is −1.3 V.

High-resolution STM images of MoSe_2_ on Au(111) show pronounced moiré superstructures within Au terraces (Figure 1d). The moiré pattern exhibits a one-third phase shift across the step edges, reflecting the ABC stacking sequence of the fcc(111) surface^45^ and indicating continuous MoSe_2_ growth across atomic steps. Figure 1e shows a close-up image of the moiré supercell. The measured MoSe_2_ lattice constant (0.329 nm)^46^ and moiré periodicity (2.3 nm)^46–48^ correspond to a commensurate 7 × 7 MoSe_2_ lattice over an 8 × 8 Au lattice (lower panel in Figure 1e). Fast Fourier transform (FFT) analysis (Figure 1f) reveals a 0° twist angle, which is further confirmed by low-energy electron diffraction (LEED) measurements (Figure 1g). These results establish a well-aligned epitaxial relationship in which the zigzag direction of MoSe_2_ is parallel to the Au⟨110⟩ direction.

Despite the structural coherence, monolayer MoSe_2_ grown by the conventional CVD method exhibits a high density of point defects (Figure 1h), with a defect density of 2.1 × 10^13^ cm^–2^, consistent with typical CVD-grown TMD materials.^49,50^ This high defect density motivates the development of an ultralow-defect growth strategy, as discussed below.

Recent STM/STS studies reveal that oxygen substitution at chalcogen sites, rather than the chalcogen vacancy, is the dominant point defect in CVD-grown TMDs.^36,51,52^ Such defects can originate from oxygen-containing precursors during growth or from postgrowth exposure to ambient.^51,53,54^ To suppress oxygen-related defects and minimize Se vacancies (V_Se_), we implemented two modifications to the CVD process: (i) an oxygen-free precursor strategy and (ii) a Se-rich cooling process.

In the oxygen-free approach, Mo is supplied through a Mo–Au alloy substrate, enabling surface-limited growth of MoSe_2_ via direct selenization in Se vapor flow,^55^ as illustrated in Figure 2a. In the Se-rich cooling approach, continuous Se vapor flow is maintained during the cooling stage to suppress V_Se_ formation. STM images of samples grown under these modified conditions (Figure 2b) reveal a dramatic reduction in the defect density compared to those grown by conventional CVD (Figure 1h). Across a total scanned area of ∼6000 nm^2^ (Figure S3), only a single defect is observed (Figure S4), corresponding to an ultralow defect density of 1.7 × 10^10^ cm^–2^.

**Figure 2:**
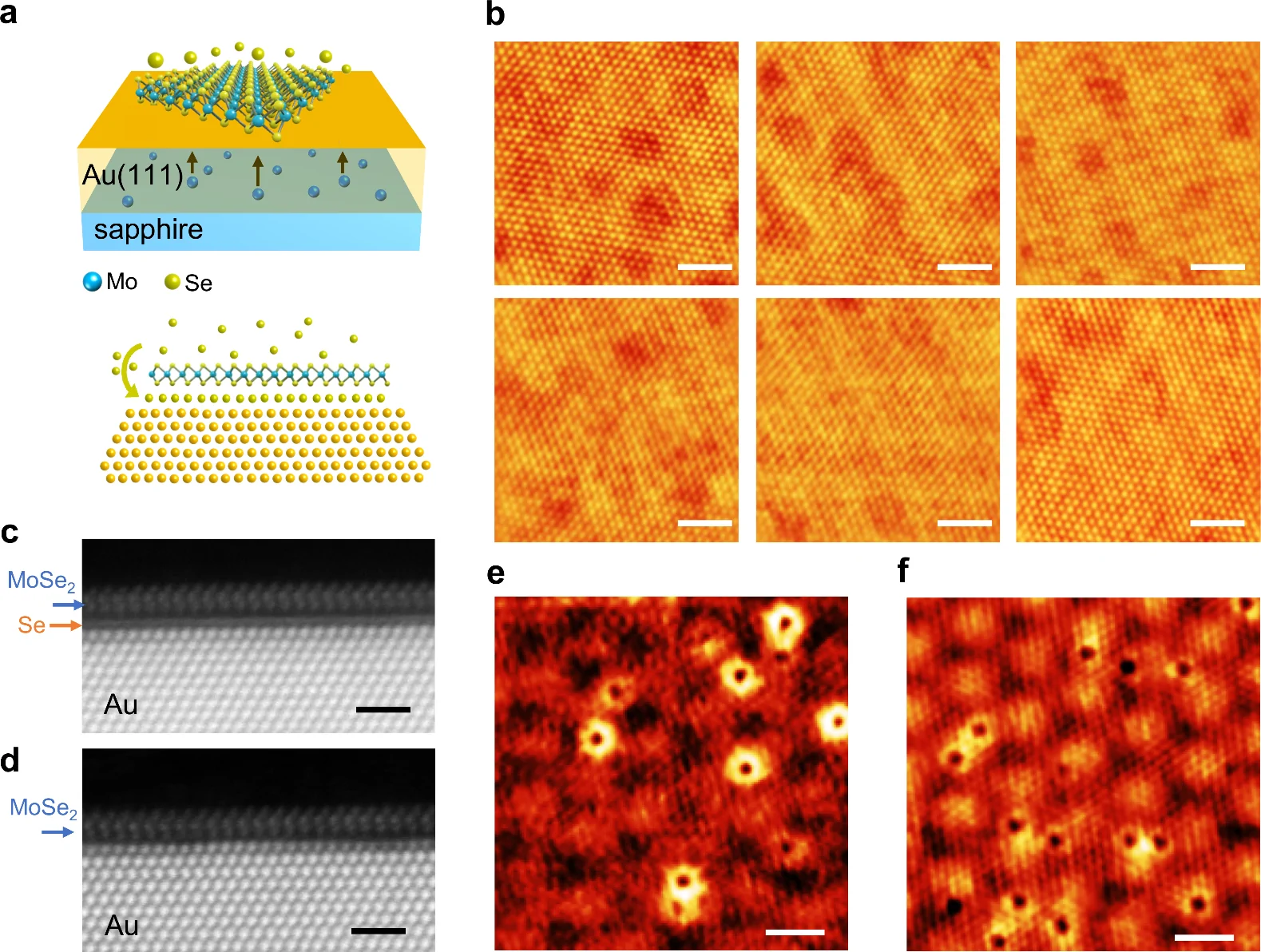
Ultralow defect density and intercalation of MoSe_2_ on Au(111). (a) Schematic of MoSe_2_ growth on a Mo–Au alloy substrate by direct selenization of Mo on the Au(111) surface (top) and Se intercalation at the MoSe_2_–Au interface under Se-rich conditions (bottom). (b) Atomically resolved STM images acquired at six randomly selected sites on MoSe_2_/Au­(111) samples grown by the oxygen-free approach and cooling under Se-rich conditions. The scan bias is −5 mV. (c and d) Cross-sectional STEM images of MoSe_2_/Au­(111) with (c) and without (d) Se intercalation. The scale bars are 1 nm. (e) STM image of MoSe_2_/Au­(111) grown by the oxygen-free approach but cooling under Se-deficient conditions. (f) STM image of MoSe_2_/Au­(111) grown by the oxygen-free approach and cooling under Se-rich conditions after 450 °C annealing in UHV. The scale bars for all STM images are 2 nm. The scan bias is −1.5 V for part e and −1.3 V for part f.

Control experiments highlight the importance of the Se-rich cooling step. Samples grown using the oxygen-free precursor but cooled without Se flow exhibit a significantly higher defect density of 4.0 × 10^12^ cm^–2^ (Figure 2e). The observed ringlike feature for these defects is consistent with V_Se_ defects, as confirmed by a comparison with simulated images (Figure S5) based on DFT. These results demonstrate that both oxygen-free growth and Se-rich cooling are essential to achieving ultralow-defect MoSe_2_ monolayers. In a Se-deficient cooling condition, abundant V_Se_ could be formed due to the low formation energy of V_Se_.^56,57^ On the other hand, the defect density of oxygen-free samples is still 5 times lower than that grown by conventional CVD, indicating that the oxygen in the precursor is still an important source of O_Se_ defects.^56,57^

Interestingly, the moiré pattern observed in conventionally grown samples disappears in those grown by using a Se-rich cooling condition (Figure 2b). The absence of a moiré pattern is attributed to Se intercalation at the MoSe_2_–Au interface during the Se-rich cooling process. Cross-sectional scanning transmission electron microscopy (STEM) images (Figure 2c,d) reveal the presence of an additional layer at the MoSe_2_–Au interface, indicating Se intercalation. This intercalated Se layer increases the interlayer spacing by ∼0.2 nm, significantly reducing the MoSe_2_–Au interaction and suppressing the moiré pattern. We noted that Se intercalation is stable in ambient conditions. In UHV, Se intercalation remains stable after 180 °C annealing for removing the surface Se capping. Importantly, this Se intercalation process is reversible: thermal annealing at 450 °C in UHV restores the moiré pattern, accompanied by the reappearance of V_Se_ (Figure 2f).

We next investigated how Au(111) modifies the electronic structure of MoSe_2_. STS measurements on MoSe_2_/Au­(111) (Figure 3a) revealed a pronounced conduction band (CB) tail extended into the band gap, which is a characteristic spectroscopic signature of MIGSs arising from hybridization between MoSe_2_ and the metallic substrate.^46–48^ Such a CB tail state has been widely observed in epitaxial TMDs on metallic surfaces and reflects intrinsic electronic coupling at the 2D–metal interface.^58–61^ In contrast, Se intercalation [MoSe_2_/Se/Au­(111)] restores sharp CB edges and suppresses the in-gap tail states (Figure 3b), indicating substantial electronic decoupling from the Au(111) substrate.

**Figure 3:**
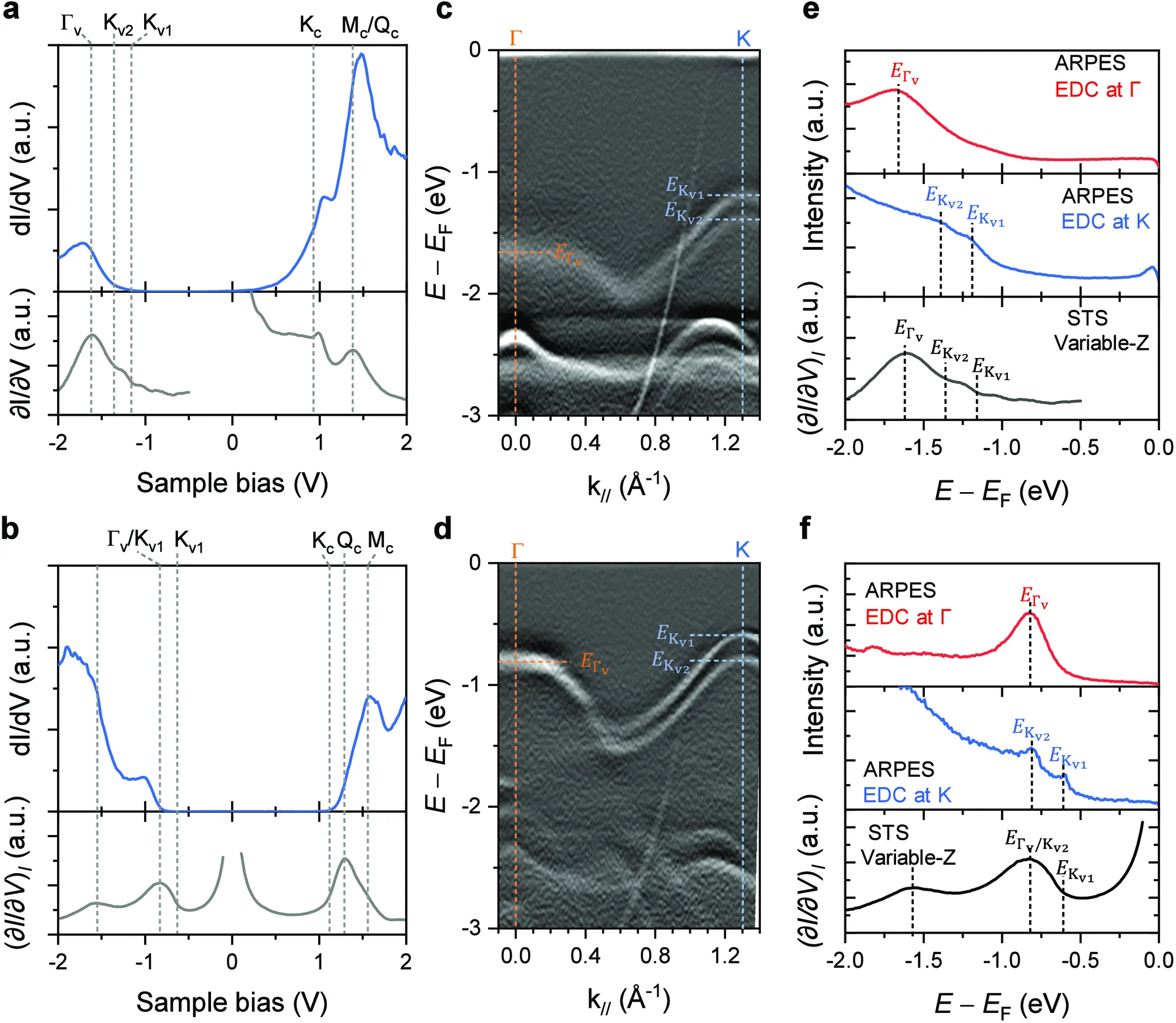
Electronic structures of MoSe_2_/Au­(111) with and without Se intercalation. STS spectra of MoSe_2_/Au­(111) (a) and MoSe_2_/Se/Au­(111) (b) measured under the constant-height mode (top) and variable-Z mode (bottom). The dashed line indicates the critical point. Second-derivative plots of ARPES spectra for the VB dispersion of MoSe_2_/Au­(111) (c) and MoSe_2_/Se/Au­(111) (d). Comparisons between the measured variable-Z (∂*I*/∂*V*)_*I*_ and the EDCs at Γ and K in ARPES for MoSe_2_/Au­(111) (e) and MoSe_2_/Se/Au­(111) (f). The EDC at Γ and K are along the orange and blue dash lines as shown in parts c and d.

Variable-Z STS measurements (lower panels in Figure 3a,b), i.e., measurements of (∂*I*/∂*V*)_*I*_ by varying the tip height at a constant current, further resolve the critical points in both the CB and VB,^62^ allowing quantitative comparison with ARPES (Figure 3c,d). Comparisons between the measured variable-Z (∂*I*/∂*V*)_*I*_ and the energy distribution curves (EDCs) at Γ and K in ARPES are shown in Figure 3e,f. For MoSe_2_/Au­(111), three VB critical points are resolved at −1.62, −1.36, and −1.16 V, which agree (within ±0.05 eV experimental error) very well with the Γ_v_, K_v1_, and K_v1_ points observed in ARPES at *E*_Γ_v__ = −1.66 eV, *E*_K_v2__ = −1.39 eV, and *E*_K_v1__ = −1.19 eV, respectively. This agreement confirms the reliability of the variable-Z STS method in resolving band extrema at the interface.

Upon Se intercalation, the ARPES results (Figure 3d) reveal pronounced upward shifts in the VB: Γ_v_ moves to *E*_Γ_v__ = −0.82 eV (Δ*E* ≈ 0.8 eV), while K_v2_ and K_v1_ move to *E*_K_v2__ = −0.81 eV and *E*_K_v1__ = −0.61 eV (Δ*E* ≈ 0.55 eV). The spectral overlap of Γ_v_ with K_v2_ and K_v1_ results in a broad variable-Z STS feature at −0.82 V, which is attributed to a mixture of the Γ_v_ and K_v2_ points. The shift at Γ_v_ indicates that this band is more sensitive to the interfacial coupling, consistent with stronger substrate-induced hybridization with the Se p_*z*_ orbitals. These systematic shifts demonstrate that the band alignment is strongly modulated by substrate interaction. We have also examined the STS spectra acquired on isolated V_Se_ defects in deintercalated MoSe_2_/Au­(111) (Figure S6). The band alignment is not shifted in comparison with that in the defect-free area (Figure 3a), which excludes residual defects as the governing mechanism behind the band alignment shifts.

To gain microscopic insight into the origin of these spectral features, we performed first-principle calculations on the full 7 × 7 MoSe_2_/8 × 8 Au(111) moiré supercell (Figure S7). The calculated unfolded band structure and projected density of states (PDOS) are presented in Figure 4a,c. We have also calculated the Se-intercalated band structure and PDOS. The main features in the PDOS of MoSe_2_/Se/Au­(111) closely resemble that of free-standing MoSe_2_ (Figure S8), with the disappearance of in-gap tail states. The band structure and PDOS of a free-standing MoSe_2_ are shown in Figure 4b,d for comparisons with that of MoSe_2_/Au­(111) in Figure 4a,c. The calculated spectra indicate that Se intercalation effectively suppresses hybridization between MoSe_2_ and Au, restoring the intrinsic band structure of monolayer MoSe_2_.

**Figure 4:**
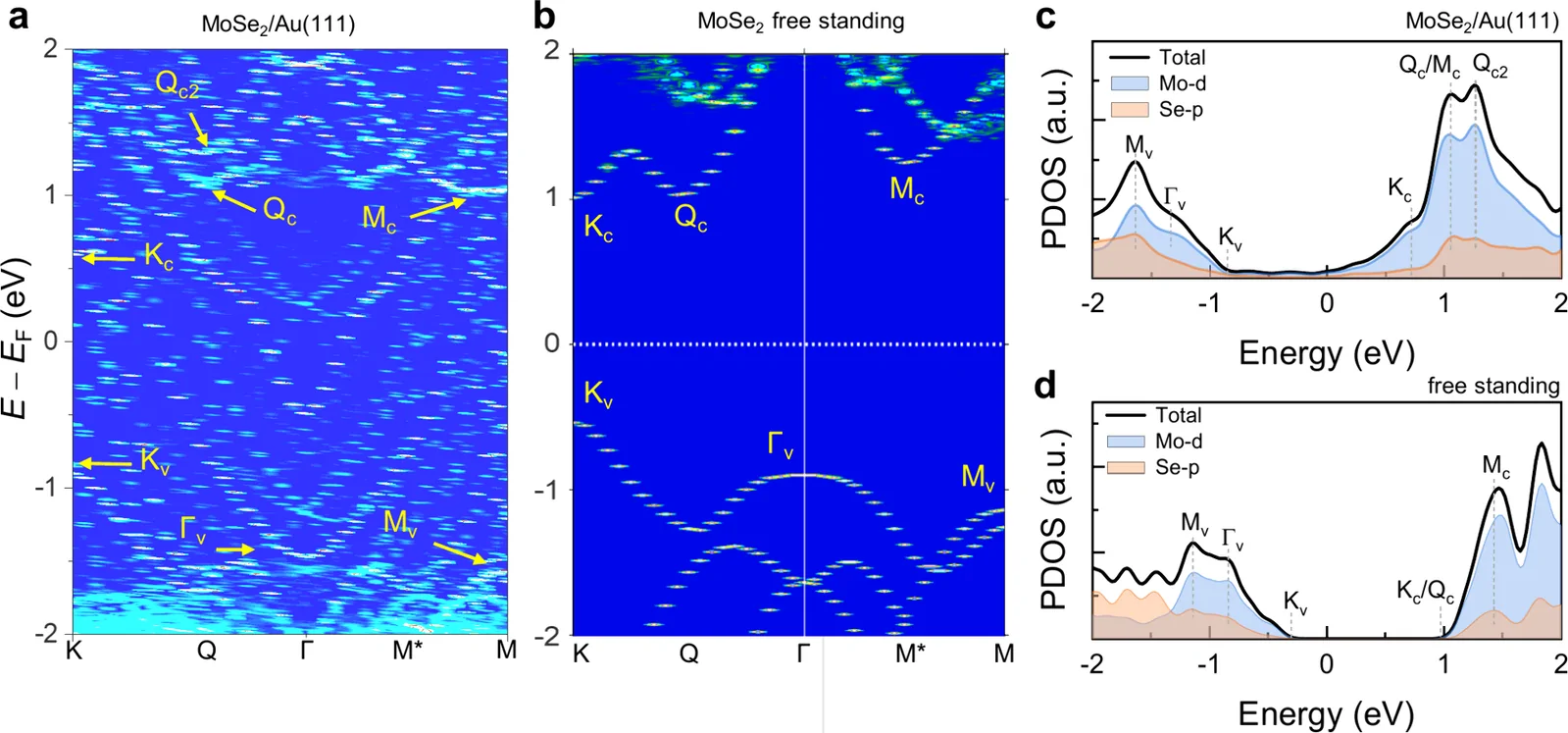
DFT calculations of the band structures and PDOSs. Calculated band structures of MoSe_2_/Au­(111) (a) and free-standing monolayer MoSe_2_ (b). Calculated PDOSs of MoSe_2_/Au­(111) (c) and free-standing monolayer MoSe_2_ (d). Shadowed areas are contributions from Mo d orbitals (blue) and Se p orbitals (orange).

The calculated PDOS reproduces the main features in the STS spectra of both MoSe_2_/Au­(111) and MoSe_2_/Se/Au­(111). For MoSe_2_/Au­(111), the PDOS in the CB shows the characteristic tail state below the CB minimum (CBM) at the K_c_ point and high-energy extrema (M_c_/Q_c_ and Q_c2_), which are consistent with the measured STS shown in Figure 3a. The comparison yields *E*_K_c__ = 0.93 eV and *E*_M_c_/Q_c__ = 1.38 eV. For the free-standing MoSe_2_, the CB tail state vanishes in the PDOS, showing a CBM at the K_c_/Q_c_ point and a high-energy extremum at the M_c_ point. Therefore, we assign the STS onset as the CBM at *E*_K_c__ = 1.12 eV and *E*_Q_c__ = 1.29 eV (*E*_M_c__ = 1.56 eV) from the peak (shoulder) in the variable-Z STS spectrum of MoSe_2_/Se/Au­(111).

Having identified the global band alignment, we next probe the local electronic structure variation across the moiré supercell in MoSe_2_/Au­(111) (Figure S9). Variable-Z STS spectra acquired on the three high-symmetry moiré sites (A–C) reveal a site-dependent modulation of the VB maximum (VBM) at Γ (Figure 5a,b). Specifically, *E*_Γ_v__ exhibits a downward shift by 0.1 eV at the A site, where the bottom Se atom resides directly above an Au atom. Spatial mapping (Figure 5c) confirms that this modulation is confined to the *E*_Γ_v__ state, while the *E*_K_v1__ and *E*_K_v2__ states remain essentially unchanged across the moiré unit cell. The site-dependent shift of *E*_Γ_v__ also accounts for broadening of the Γ feature in the ARPES spectra, which averages over the moiré superstructure. The unchanged K-point states indicate that the global band alignment and Fermi level position are not locally modulated by the moiré structure, in contrast to the local work function modulation commonly observed in other 2D/metal systems due to spatial charge redistribution within different moiré sites.^63–67^ DFT calculation of the Hartree potential in the MoSe_2_/Au­(111) moiré supercell (Figure S10) also shows a small variation (∼22 meV) across the moiré supercell, indicating a negligible variation in the local charge distribution (Figure S11).

**Figure 5:**
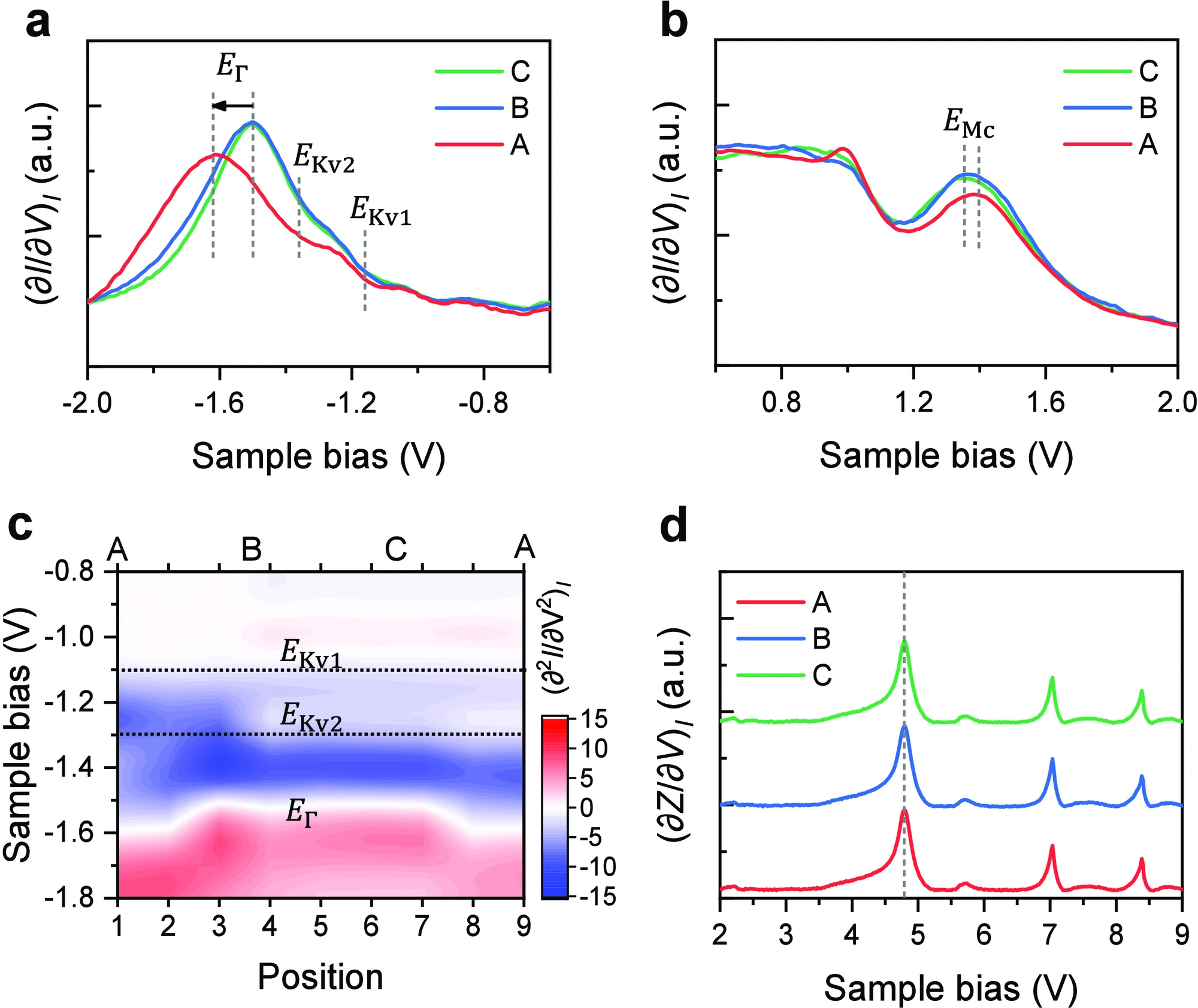
Moiré-modulated local electronic structures. Variable-Z spectra (∂*I*/∂*V*)_*I*_ of the VB (a) and CB (b) acquired at the three high-symmetry moiré sites A–C. (c) Contour plot of the first derivative variable-Z spectra (∂^2^ *I*/∂*V* ^2^)_*I*_ across the moiré supercell. (d) FER spectra acquired at the A–C sites.

To directly probe possible work function variations, we performed FER spectroscopy across the moiré supercell by measurements of (∂*I*/∂*V*)_*I*_ spectra at large sample biases. The first FER peak, which provides a reliable approximation of the local work function,^63,65^ remains unchanged at different moiré sites (Figure 5d). These measurements demonstrate that while local hybridization modulates specific band edges, the interfacial dipole and overall work function remain spatially uniform. The FLP observed in MoSe_2_/Au­(111) is therefore governed by a global interface effect rather than local moiré-induced potential variations.

The ultralow-defect MoSe_2_/Au­(111) epitaxial interface provides a model system for isolating intrinsic FLPs without complications from extrinsic DIGSs. Theoretical studies have proposed that intrinsic FLP at the 2D–metal interface arises from the interface dipoles induced by charge redistribution and the associated formation of MIGSs.^25^ Such interface dipoles modify the effective metal work function and, thereby, the SBH.

Within the modified Schottky–Mott framework, the SBH for electrons (Φ_Bn_) and holes (Φ_Bp_) can be determined by

$$\Phi_{\mathrm{Bn}}=W_{m}-\chi_{s}-\Delta ,\mathrm{and},\Phi_{\mathrm{Bp}}=I_{s}-W_{m}+\Delta$$

where *W*_m_ is the metal work function, χ_s_ and *I*_s_ are the electron affinity and ionization energy of the semiconductor, and Δ is the interface potential step induced by the interface dipoles, which is equivalent to the work function difference between the metal *W*_m_ and the metal covered with 2D semiconductor *W*_ms_, i.e., Δ = *W*_m_ – *W*_ms_, as schematically illustrated in Figure 6a,b.

**Figure 6:**
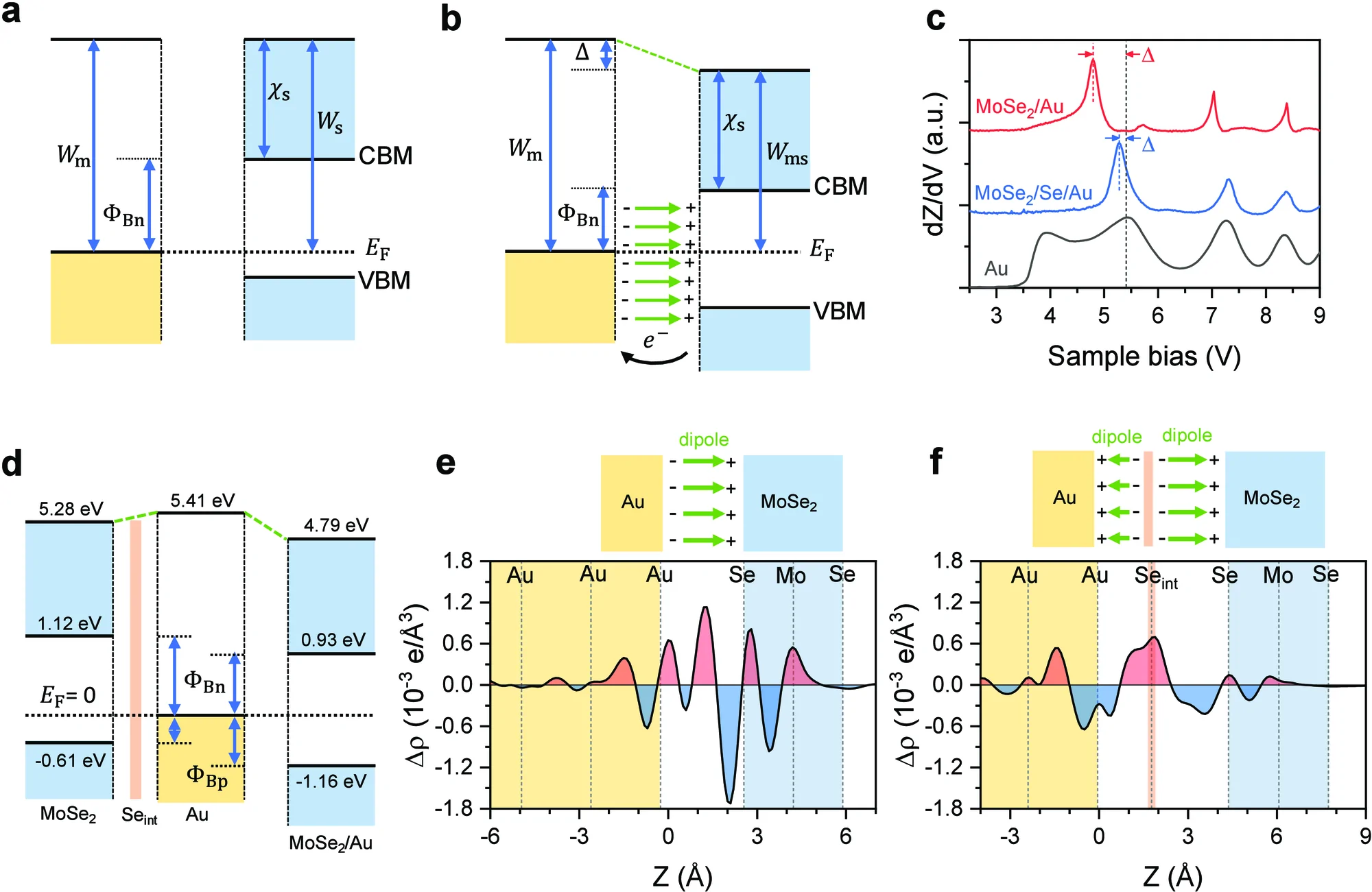
Work function lowering by interface dipoles. Schematic of the band alignments between the metal and semiconductor according to Schottky–Mott without (a) and with (b) interface dipoles. (c) FER spectra of MoSe_2_/Au­(111), MoSe_2_/Se/Au­(111), and bare Au(111). (d) Schematic of the band alignment for MoSe_2_/Au­(111) and MoSe_2_/Se/Au­(111). Plane-averaged charge-density difference Δρ­(*z*) along the *z* direction (normal to the interface) for MoSe_2_/Au­(111) (e) and MoSe_2_/Se/Au­(111) (f).

FER measurements (Figure 6c) reveal that the work function decreases from *W*_Au_ = 5.41 eV for bare Au(111) to *W*_MoSe_2_/Au_ = 4.79 eV after MoSe_2_ deposition, corresponding to a substantial reduction of 0.62 eV. Combining these values with the band edges determined by STS and ARPES, we extract *I*_s_ = 5.95 eV and χ_s_ = 3.86 eV for monolayer MoSe_2_/Au­(111) (Figure 6d).^68–71^ The resulting SBHs, Φ_Bn_ = 0.93 eV and Φ_Bp_ = 1.16 eV, are nearly symmetric, indicating a FLP at about midgap with a slightly n-type character. We use the measured Φ_Bn_ and χ_s_ to give a rough estimation for the pinning factor *S* of the MoSe_2_/Au­(111) interface by assuming Φ_Bn_ = 0 at *W*_m_ = χ_s_ according to the Schottky–Mott rule (Figure S12). We obtain a moderate pinning factor of *S* = 0.6, which is distinct from the strong pinning factor (*S* = 0.1–0.3) for interfaces affected by extrinsic defects,^20–22^ implying that the intrinsic FLP is weaker than the DIGS-induced pinning. Upon Se intercalation, the work function increases to *W*_MoSe_2_/Se/Au_ = 5.28 eV, approaching that of bare Au(111). Correspondingly, the Fermi level shifts toward the VBM, yielding Φ_Bn_ = 1.12 eV and Φ_Bp_ = 0.61 eV and a pronounced p-type character. The estimated pinning factor for the Se-intercalated interface is *S* = 0.9, which is close to an unpinned interface. The restoration of the work function demonstrates that intercalation suppresses the interface dipole and weakens metal–semiconductor hybridization.

First-principles calculations of the charge-density difference Δρ­(*z*) provide microscopic insight into this dipole modulation. Parts e and f of Figure 6 show the plane-averaged electron-density differences Δρ­(*z*) along the *z* direction perpendicular to the interfaces. The electron-density difference is defined as Δρ = ρ_ms_ – ρ_m_ – ρ_s_, where ρ_ms_, ρ_m_, and ρ_s_ are the electron densities in the composite Au–MoSe_2_ interface, the isolated Au metal, and the free-standing MoSe_2_. For MoSe_2_/Au­(111) (Figure 6e), electron accumulation (red in Δρ) close to the first Au layer and electron depletion (blue in Δρ) near the interfacial Se layer create an interface dipole pointing from Au to MoSe_2_,^25^ producing a large potential drop of Δ = 0.49 eV, consistent with the experimentally observed work function reduction. In contrast, Se intercalation (Figure 6f) introduces an additional charge redistribution layer that forms two opposite dipole layers, substantially reducing the net dipole strength and restoring the metal work function. These findings demonstrate that the observed FLP and work function suppression arise from dipole formation rather than extrinsic defect states. The ability to reversibly modulate the dipole strength via intercalation provides a direct route to controlling the band alignment at 2D–metal interfaces.

In conclusion, we demonstrate that FLP persists at an ultralow-defect epitaxial 2D–metal interface, providing direct experimental evidence for its intrinsic origin in van der Waals contacts. Using a structurally pristine MoSe_2_/Au­(111) platform with minimized DIGSs, we directly probe intrinsic electronic coupling at a 2D–metal interface. Symmetric SBHs and a pronounced work function lowering remain even in the near absence of interfacial disorder. Combined spectroscopic measurements and first-principles calculations consistently support a mechanism in which MIGSs generate interfacial dipoles that fix the Fermi level near midgap. Beyond establishing intrinsic pinning, we further show that Se intercalation reversibly modulates the interfacial dipole strength, enabling systematic tuning of the work function and band alignment. This controlled depinning distinguishes intrinsic interfacial coupling from disorder-driven effects. Our results clarify the intrinsic origin of FLP and provide a viable scheme for controlling the band alignment at 2D–metal interfaces.

## Supplementary Material



## Notes

Materials synthesis, characterization and computational details, formation of the Au(111) surface, preparation of Au and Mo–Au alloy substrates, STM images of ultralow-defect MoSe_2_/Au­(111), defect characterization in ultralow-defect MoSe_2_, experimental and simulated STM images of defects, STS spectra acquired on an isolated V_Se_ and on defect-free regions, calculation model for MoSe_2_ on a Au(111) supercell, calculated PDOS, atomically resolved STM image of the moiré supercell, calculated Hartree potential across the moiré supercell, charge-density difference in a moiré supercell of MoSe_2_/Au­(111), and estimation of the pinning factor *S* (PDF)

## References

[1] Chhowalla M., Jena D., Zhang H. (2016). Two-dimensional semiconductors for
transistors. Nat. Rev. Mater..

[2] Long M., Wang P., Fang H., Hu W. (2019). Progress, challenges,
and opportunities for 2D material based photodetectors. Adv. Funct. Mater..

[3] Das S., Sebastian A., Pop E., McClellan C. J., Franklin A. D., Grasser T., Knobloch T., Illarionov Y., Penumatcha A. V., Appenzeller J., Chen Z., Zhu W., Asselberghs I., Li L.-J., Avci U. E., Bhat N., Anthopoulos T. D., Singh R. (2021). Transistors based on two-dimensional
materials for future integrated circuits. Nat.
Electron..

[4] Liu Y., Duan X., Shin H.-J., Park S., Huang Y., Duan X. (2021). Promises and
prospects of two-dimensional transistors. Nature.

[5] O’Brien K. P., Naylor C. H., Dorow C., Maxey K., Penumatcha A. V., Vyatskikh A., Zhong T., Kitamura A., Lee S., Rogan C., Mortelmans W., Kavrik M. S., Steinhardt R., Buragohain P., Dutta S., Tronic T., Clendenning S., Fischer P., Putna E. S., Radosavljevic M., Metz M., Avci U. (2023). Process integration and future outlook
of 2D transistors. Nat. Commun..

[6] Li M.-Y., Su S.-K., Wong H.-S. P., Li L.-J. (2019). How 2D semiconductors
could extend Moore’s law?. Nature.

[7] Cao W., Bu H., Vinet M., Cao M., Takagi S., Hwang S., Ghani T., Banerjee K. (2023). The future
transistors. Nature.

[8] Kim K. S., Kwon J., Ryu H., Kim C., Kim H., Lee E.-K., Lee D., Seo S., Han N. M., Suh J. M., Kim J., Song M.-K., Lee S., Seol M., Kim J. (2024). The future of two-dimensional semiconductors
beyond Moore’s law. Nat. Nanotechnol..

[9] Wu P., Zhang T., Zhu J., Palacios T., Kong J. (2024). 2D materials
for logic device scaling. Nat. Mater..

[10] Kang J., Liu W., Sarkar D., Jena D., Banerjee K. (2014). Computational study
of metal contacts to monolayer transition-metal dichalcogenide semiconductors. Phys. Rev. X.

[11] Allain A., Kang J., Banerjee K., Kis A. (2015). Electrical contacts
to two-dimensional semiconductors. Nat. Mater..

[12] Schulman D. S., Arnold A. J., Das S. (2018). Contact Engineering
for 2D Materials
and Devices. Chem. Soc. Rev..

[13] Huang L., Tao L., Gong K., Li Y., Dong H., Wei Z., Li J. (2017). Role of defects in
enhanced Fermi level pinning at interfaces between
metals and transition metal dichalcogenides. Phys. Rev. B.

[14] Zhang P., Zhang Y., Wei Y., Jiang H., Wang X., Gong Y. (2020). Contact engineering
for two-dimensional semiconductors. J. Semicond..

[15] Shen P.-C., Su C., Lin Y., Chou A.-S., Cheng C.-C., Park J.-H., Chiu M.-H., Lu A.-Y., Tang H.-L., Tavakoli M. M., Pitner G., Ji X., Cai Z., Mao N., Wang J., Tung V., Li J., Bokor J., Zettl A., Wu C.-I., Palacios T., Li L.-J., Kong J. (2021). Ultralow contact resistance between
semimetal and monolayer semiconductors. Nature.

[16] Li W., Gong X., Yu Z., Ma L., Sun W., Gao S., Köroǧlu Ç., Wang W., Liu L., Li T., Ning H., Fan D., Xu Y., Tu X., Xu T., Sun L., Wang W., Lu J., Ni Z., Li J., Duan X., Wang P., Nie Y., Qiu H., Shi Y., Pop E., Wang J., Wang X. (2023). Approaching the quantum
limit in two-dimensional semiconductor contacts. Nature.

[17] Li S., Chen J., He X., Zheng Y., Yu C., Lu H. (2023). Comparative study of
the micro-mechanism of charge redistribution
at metal-semiconductor and semimetal-semiconductor interfaces: Pt­(Ni)-MoS_2_ and Bi-MoS_2_(WSe_2_) as the prototype. Appl. Surf. Sci..

[18] Wang X., Hu Y., Kim S. Y., Addou R., Cho K., Wallace R. M. (2023). Origins
of Fermi Level Pinning for Ni and Ag Metal Contacts on Tungsten Dichalcogenides. ACS Nano.

[19] Jiang J., Xu L., Du L., Li L., Zhang G., Qiu C., Peng L.-M. (2024). Yttrium-doping-induced
metallization of molybdenum
disulfide for ohmic contacts in two-dimensional transistors. Nat. Electron..

[20] Kim C., Moon I., Lee D., Choi M. S., Ahmed F., Nam S., Cho Y., Shin H.-J., Park S., Yoo W. J. (2017). Fermi level
pinning at electrical metal contacts of monolayer molybdenum dichalcogenides. ACS Nano.

[21] Bampoulis P., van Bremen R., Yao Q., Poelsema B., Zandvliet H. J. W., Sotthewes K. (2017). Defect dominated charge transport and Fermi level pinning
in MoS_2_/metal contacts. ACS Appl.
Mater. Interfaces.

[22] Sotthewes K., van Bremen R., Dollekamp E., Boulogne T., Nowakowski K., Kas D., Zandvliet H. J. W., Bampoulis P. (2019). Universal
Fermi-level pinning in transition-metal dichalcogenides. J. Phys. Chem. C.

[23] Liu X., Choi M. S., Hwang E., Yoo W. J., Sun J. (2022). Fermi Level
Pinning Dependent 2D Semiconductor. Adv. Mater..

[24] Chen W., Santos E. J. G., Zhu W., Kaxiras E., Zhang Z. (2013). Tuning the
Electronic and Chemical Properties of Monolayer MoS_2_ Adsorbed
on Transition Metal Substrates. Nano Lett..

[25] Gong C., Colombo L., Wallace R. M., Cho K. (2014). The Unusual Mechanism
of Partial Fermi Level Pinning at Metal-MoS_2_ Interfaces. Nano Lett..

[26] Liu Y., Stradins P., Wei S.-H. (2016). Van der
Waals Metal-Semiconductor
Junction: Weak Fermi Level Pinning Enables Effective Tuning of Schottky
Barrier. Sci. Adv..

[27] Farmanbar M., Brocks G. (2016). First-principles study
of van der Waals interactions
and lattice mismatch at MoS_2_/metal interfaces. Phys. Rev. B.

[28] Min K.-A., Park J., Wallace R. M., Cho K., Hong S. (2017). Reduction
of Fermi Level Pinning at Au-MoS_2_ Interfaces by Atomic
Passivation on Au Surface. 2D Mater..

[29] Huang L., Li B., Zhong M., Wei Z., Li J. (2017). Tunable Schottky Barrier
at MoSe_2_/Metal Interfaces with a Buffer Layer. J. Phys. Chem. C.

[30] Heine V. (1965). Theory of
surface states. Phys. Rev..

[31] Tersoff J. (1984). Schottky barrier
heights and the continuum of gap states. Phys.
Rev. Lett..

[32] Mönch W. (1990). On the physics
of metal-semiconductor interfaces. Rep. Prog.
Phys..

[33] Mleczko M. J., Yu A. C., Smyth C. M., Chen V., Shin Y. C., Chatterjee S., Tsai Y.-C., Nishi Y., Wallace R. M., Pop E. (2019). Contact Engineering High-Performance n-Type MoTe_2_ Transistors. Nano Lett..

[34] Wu R. J., Udyavara S., Ma R., Wang Y., Chhowalla M., Birol T., Koester S. J., Neurock M., Mkhoyan K. A. (2019). Visualizing
the Metal-MoS_2_ Contacts in Two-Dimensional Field-Effect
Transistors with Atomic Resolution. Phys. Rev.
Mater..

[35] Smyth C. M., Addou R., Hinkle C. L., Wallace R. M. (2019). Origins of Fermi-Level
Pinning between Molybdenum Dichalcogenides (MoSe_2_, MoTe_2_) and Bulk Metal Contacts: Interface Chemistry and Band Alignment. J. Phys. Chem. C.

[36] Schuler B., Lee J.-H., Kastl C., Cochrane K. A., Chen C. T., Refaely-Abramson S., Yuan S., van Veen E., Roldán R., Borys N. J., Koch R. J., Aloni S., Schwartzberg A. M., Ogletree D. F., Neaton J. B., Weber-Bargioni A. (2019). How Substitutional
Point Defects in Two-Dimensional WS_2_ Induce Charge Localization,
Spin-Orbit Splitting, and Strain. ACS Nano.

[37] Trainer D. J., Nieminen J., Bobba F., Wang B., Xi X., Bansil A., Iavarone M. (2022). Visualization of Defect Induced In-Gap
States in Monolayer MoS_2_. npj 2D
Mater. Appl..

[38] Aliyar T., Ma H., Krishnan R., Singh G., Chong B. Q., Wang Y., Verzhbitskiy I., Yu Wong C. P., Johnson Goh K. E., Shen Z. X., Koh T. S., Rahman R., Weber B. (2024). Symmetry Breaking
and Spin-Orbit Coupling for Individual Vacancy-Induced In-Gap States
in MoS_2_ Monolayers. Nano Lett..

[39] Schauble K., Zakhidov D., Yalon E., Deshmukh S., Grady R. W., Cooley K. A., McClellan C. J., Vaziri S., Passarello D., Mohney S. E., Toney M. F., Sood A. K., Salleo A., Pop E. (2020). Uncovering the Effects of Metal Contacts on Monolayer MoS_2_. ACS Nano.

[40] Wang X., Hu Y., Kim S. Y., Cho K., Wallace R. M. (2024). Mechanism of Fermi
Level Pinning for Metal Contacts on Molybdenum Dichalcogenide. ACS Appl. Mater. Interfaces.

[41] Liu Y., Guo J., Zhu E., Liao L., Lee S.-J., Ding M., Shakir I., Gambin V., Huang Y., Duan X. (2018). Approaching
the Schottky-Mott Limit in Van der Waals Metal-Semiconductor Junctions. Nature.

[42] Jung Y., Choi M. S., Nipane A., Borah A., Kim B., Zangiabadi A., Taniguchi T., Watanabe K., Yoo W. J., Hone J., Teherani J. T. (2019). Transferred Via Contacts as a Platform
for Ideal Two-Dimensional Transistors. Nat.
Electron..

[43] Wang Y., Kim J. C., Wu R. J., Martinez J., Song X., Yang J., Zhao F., Mkhoyan A., Jeong H. Y., Chhowalla M. (2019). Van der Waals Contacts between Three-Dimensional
Metals
and Two-Dimensional Semiconductors. Nature.

[44] Wang Y., Kim J. C., Li Y., Ma K. Y., Hong S., Kim M., Shin H. S., Jeong H. Y., Chhowalla M. (2022). P-Type Electrical
Contacts for 2D Transition-Metal Dichalcogenides. Nature.

[45] Zhang H., Holbrook M., Cheng F., Nam H., Liu M., Pan C.-R., West D., Zhang S., Chou M.-Y., Shih C.-K. (2021). Epitaxial Growth of Two-Dimensional Insulator Monolayer
Honeycomb BeO. ACS Nano.

[46] Lu J., Bao D.-L., Qian K., Zhang S., Chen H., Lin X., Du S.-X., Gao H.-J. (2017). Identifying and Visualizing the Edge
Terminations of Single-Layer MoSe_2_ Island Epitaxially Grown
on Au(111). ACS Nano.

[47] Yasuda S., Takahashi R., Osaka R., Kumagai R., Miyata Y., Okada S., Hayamizu Y., Murakoshi K. (2017). Out-of-Plane
Strain Induced in a Moiré Superstructure of Monolayer MoS_2_ and MoSe_2_ on Au(111). Small.

[48] Cheng F., Hu Z., Xu H., Shao Y., Su J., Chen Z., Ji W., Loh K. P. (2019). Interface Engineering of Au(111) for the Growth of
1T’-MoSe_2_. ACS Nano.

[49] Hong J., Hu Z., Probert M., Li K., Lv D., Yang X., Gu L., Mao N., Feng Q., Xie L., Zhang J., Wu D., Zhang Z., Jin C., Ji W., Zhang X., Yuan J., Zhang Z. (2015). Exploring Atomic Defects in Molybdenum
Disulphide Monolayers. Nat. Commun..

[50] Picker J., Schaal M., Gan Z., Gruenewald M., Neumann C., George A., Otto F., Forker R., Fritz T., Turchanin A. (2023). Structural
and electronic properties
of MoS_2_ and MoSe_2_ monolayers grown by chemical
vapor deposition on Au(111). Nanoscale Adv..

[51] Barja S., Refaely-Abramson S., Schuler B., Qiu D. Y., Pulkin A., Wickenburg S., Ryu H., Ugeda M. M., Kastl C., Chen C., Hwang C., Schwartzberg A., Aloni S., Mo S.-K., Ogletree D. F., Crommie M. F., Yazyev O. V., Louie S. G., Neaton J. B., Weber-Bargioni A. (2019). Identifying
Substitutional Oxygen as a Prolific Point Defect in Monolayer Transition
Metal Dichalcogenides. Nat. Commun..

[52] Zheng Y. J., Chen Y., Huang Y. L., Gogoi P. K., Li M.-Y., Li L.-J., Trevisanutto P. E., Wang Q., Pennycook S. J., Wee A. T. S., Quek S. Y. (2019). Point Defects
and Localized Excitons
in 2D WSe_2_. ACS Nano.

[53] Petö J., Ollár T., Vancsó P., Popov Z. I., Magda G. Z., Dobrik G., Hwang C., Sorokin P. B., Tapasztó L. (2018). Spontaneous
Doping of the Basal Plane of MoS_2_ Single Layers through
Oxygen Substitution under Ambient Conditions. Nat. Chem..

[54] Grønborg S. S., Thorarinsdottir K., Kyhl L., Rodriguez-Fernández J., Sanders C. E., Bianchi M., Hofmann P., Miwa J. A., Ulstrup S., Lauritsen J. V. (2019). Basal Plane Oxygen Exchange of Epitaxial
MoS_2_ Without Edge Oxidation. 2D Mater..

[55] Shivayogimath A., Thomsen J. D., Mackenzie D. M. A., Geisler M., Stan R.-M., Holt A. J., Bianchi M., Crovetto A., Whelan P. R., Carvalho A., Neto A. H. C., Hofmann P., Stenger N., Bøggild P., Booth T. J. (2019). A Universal Approach for the Synthesis
of Two-Dimensional Binary Compounds. Nat. Commun..

[56] Azcatl A., Santosh K. C., Peng X., Lu N., McDonnell S., Qin X., de Dios F., Addou R., Kim J., Kim M. J., Cho K., Wallace R. M. (2015). HfO_2_ on
UV-O_3_ Exposed Transition
Metal Dichalcogenides: Interfacial Reactions Study. 2D Mater..

[57] Shu H., Zhou D., Li F., Cao D., Chen X. (2017). Defect Engineering
in MoSe_2_ for the Hydrogen Evolution Reaction: From Point
Defects to Edges. ACS Appl. Mater. Interfaces.

[58] Sørensen S. G., Füchtbauer H. G., Tuxen A. K., Walton A. S., Lauritsen J. V. (2014). Structure
and Electronic Properties of In Situ Synthesized Single-Layer MoS_2_ on a Gold Surface. ACS Nano.

[59] Addou R., McDonnell S., Barrera D., Guo Z., Azcatl A., Wang J., Zhu H., Hinkle C. L., Quevedo-Lopez M., Alshareef H. N., Colombo L., Hsu J. W. P., Wallace R. M. (2015). Impurities
and Electronic Property Variations of Natural MoS_2_ Crystal
Surfaces. ACS Nano.

[60] Bruix A., Miwa J. A., Hauptmann N., Wegner D., Ulstrup S., Grønborg S. S., Sanders C. E., Dendzik M., Grubisic
Cabo A., Bianchi M., Lauritsen J. V., Khajetoorians A. A., Hammer B.ør., Hofmann P. (2016). Single-Layer MoS_2_ on Au(111):
Band Gap Renormalization and Substrate Interaction. Phys. Rev. B.

[61] Krane N., Lotze C., Franke K. J. (2018). Moiré Structure
of MoS_2_ on Au(111): Local Structural and Electronic Properties. Surf. Sci..

[62] Zhang C., Chen Y., Johnson A., Li M.-Y., Li L.-J., Mende P. C., Feenstra R. M., Shih C.-K. (2015). Probing
Critical
Point Energies of Transition Metal Dichalcogenides: Surprising Indirect
Gap of Single Layer WSe_2_. Nano Lett..

[63] Joshi S., Ecija D., Koitz R., Iannuzzi M., Seitsonen A. P., Hutter J., Sachdev H., Vijayaraghavan S., Bischoff F., Seufert K., Barth J. V., Auwärter W. (2012). Boron Nitride
on Cu(111): An Electronically Corrugated Monolayer. Nano Lett..

[64] Wang B., Caffio M., Bromley C., Früchtl H., Schaub R. (2010). Coupling Epitaxy, Chemical Bonding, and Work Function
at the Local Scale in Transition Metal-Supported Graphene. ACS Nano.

[65] Schulz F., Drost R., Hämäläinen S. K., Demonchaux T., Seitsonen A. P., Liljeroth P. (2014). Epitaxial
Hexagonal Boron Nitride on Ir(111): A Work Function Template. Phys. Rev. B.

[66] Zhang Q., Chen Y., Zhang C., Pan C.-R., Chou M.-Y., Zeng C., Shih C.-K. (2016). Bandgap Renormalization
and Work
Function Tuning in MoSe_2_/hBN/Ru­(0001) Heterostructures. Nat. Commun..

[67] Zhang Q., Yu J., Ebert P., Zhang C., Pan C.-R., Chou M.-Y., Shih C.-K., Zeng C., Yuan S. (2018). Tuning Band Gap and
Work Function Modulations in Monolayer hBN/Cu(111) Heterostructures
with Moiré Patterns. ACS Nano.

[68] Aretouli K. E., Tsipas P., Tsoutsou D., Marquez-Velasco J., Xenogiannopoulou E., Giamini S. A., Vassalou E., Kelaidis N., Dimoulas A. (2015). Two-Dimensional Semiconductor HfSe_2_ and
MoSe_2_/HfSe_2_ Van der Waals Heterostructures by
Molecular Beam Epitaxy. Appl. Phys. Lett..

[69] Xiao J., Zhang L., Zhou H., Shao Z., Liu J., Zhao Y., Li Y., Liu X., Xie H., Gao Y., Sun J.-T., Wee A. T. S., Huang H. (2020). Type-II Interface Band
Alignment in the vdW PbI_2_-MoSe_2_ Heterostructure. ACS Appl. Mater. Interfaces.

[70] Kim H.-G., Choi H. J. (2021). Thickness dependence
of work function, ionization energy,
and electron affinity of Mo and W dichalcogenides from DFT and GW
calculations. Phys. Rev. B.

[71] Li C., Wu Z., Zhang C., Peng S., Han J., He M., Dong X., Gou J., Wang J., Jiang Y. (2023). Self-Powered
Photodetector with High Performance Based on All-2D NbSe_2_/MoSe_2_ Van der Waals Heterostructure. Adv. Opt. Mater..

