# Supplementary material for: Intrinsic Fermi Level Pinning and Reversible Depinning at Ultralow-Defect Epitaxial MoSe2/Au(111) Interfaces
Source: Nano Lett. 2026 Sep 20;26(38):12675–84. doi: 10.1021/acs.nanolett.6c02384 (PMC13632591; doi:10.1021/acs.nanolett.6c02384)
Supplement: Supplementary file 1 [file nl6c02384_si_001.pdf]

Supporting Information for

Intrinsic Fermi level pinning and reversible depinning at ultralow-defect epitaxial $MoSe_2$/Au(111) interfaces

Guan-Hao Chen[1,2^], Cheng-Rong Hsing[3,4^], Li-Syuan Lu[1,2], Po-Sen Mao,[1,5] Pei-Yu Chuang[6], Yi Chou[1], Yu-Shiuan Su[1,7], Chih-Pin Lin[8], Tuo-Hung Hou[8], Juhn-Jong Lin[1], Yi-Chia Chou[9], Cheng-Maw Cheng[1,6,10], Ching-Ming Wei[11*], Chun-Liang Lin,[1*] Wen-Hao Chang[1,2,5,12*]

[1]*Department of Electrophysics, National Yang Ming Chiao Tung University, Hsinchu 30010, Taiwan*
[2]*Research Center for Applied Sciences, Academia Sinica, Taipei 11529, Taiwan*
[3]*Division of Natural Science, Center for General Education, Chang Gung University, Taoyuan City 33302, Taiwan.*
[4]*Department of Artificial Intelligence, Chang Gung University, Taoyuan City 33302, Taiwan*
[5]*Research Center for Critical Issues, Academia Sinica, Tainan 711010, Taiwan*
[6]*National Synchrotron Radiation Research Center, Hsinchu 300, Taiwan*
[7]*Taiwan Instrument Research Institute, Hsinchu 300092, Taiwan*
[8]*Department of Electronics Engineering and Institute of Electronics, National Yang Ming Chiao Tung University, Hsinchu, 30010, Taiwan*
[9]*Department of Materials Science and Engineering, National Taiwan University, Taipei 10617, Taiwan*
[10]*Department of Physics, National Sun Yat-sen University, Kaohsiung 80424, Taiwan*
[11]*Institute of Atomic and Molecular Sciences, Academia Sinica, Taipei 106923, Taiwan*
[12]*Center for Emergent Functional Matter Science (CEFMS), National Yang Ming Chiao Tung University, Hsinchu 30010, Taiwan*

*To whom correspondence should be addressed: whchang@nycu.edu.tw (W.-H.C.), clin@nycu.edu.tw (C.-L.L) and cmw@gate.sinica.edu.tw (C.-M.W)
^Equal contribution

Methods

Materials synthesis.

Preparation of Au substrates. For the single-crystal Au(111) substrates, a thin Te layer was first deposited on a c-plane sapphire substrate followed by depositing a thick Au layer using sputtering. The samples were then thermally annealed at 1,000 °C for 10 mins to form a single-crystal Au(111) surface. For samples annealed at 850 °C, irregular polycrystalline grains remain on the surface (**Figure S1**). Using a thin Te insert layer here is crucial for lowering the melting temperature of gold,[1] facilitating the crystallization of the Au(111) surface (**Figure S2**). For the Mo-Au alloy substrates, a 3-nm Te layer was first sputtered on c-plane sapphire substrates at room temperature, followed by Mo deposition at 200 ℃ in the Ar environment. Finally, a 700-1000 nm Au layer was deposited at room temperature by using either sputtering or electron-beam evaporation. After depositions, the samples were thermally annealed at 1,000 °C for 10 mins to form a single-crystal Au(111) surface.

Oxygen-free $MoSe_2$ growth. Monolayer $MoSe_2$ was grown by exposing the Mo-Au alloy substrate in Se vapor flow in a one-inch horizontal furnace equipped with two heating zones. The as-prepared Mo-Au alloy substrate was positioned at the main heating zone, while Se powder (Sigma-Aldrich, 99.995%) was placed in the upstream heating zone for supplying Se vapor. After evacuating the furnace, a 10% H_2/Ar mixture gas was introduced, and the main heating zone was ramped to 1,000 ℃ for 10 minutes to anneal the Mo-Au alloy substrate to form a single-crystal Au(111) surface. Subsequently, the temperature was lowered to 880 ℃ and the Se temperature was raised to 300 ℃ for $MoSe_2$ growth. The growth was proceeding at 880 ℃ for 10 minutes at a base pressure of 30 torr. Due to the low solubility of Se in Au, the reaction is surface-limited,[2] occurring at the solid-gas interface, and results in the epitaxial growth of monolayer $MoSe_2$ on Au(111). After growth, the system was cooled down naturally to room temperature. The $MoSe_2$ surface was capped with a Se layer in the furnace by supplying Se vapor flow when the substrate temperature was cooled down to below 100 °C. This step is crucial for protecting the sample surface from exposure to air for subsequent STM characterizations.

Conventional CVD-grown monolayer $MoSe_2$. Monolayer $MoSe_2$ was synthesized on Au(111) substrates using MoO_2 powders (Sigma-Aldrich, 99%) and Se powders (Sigma-Aldrich, 99.995%) as precursors. The growth was proceeding in a one-inch horizontal furnace equipped with two heating zones. The MoO_2 powder and the substrate were placed at the main heating zone, while the Se powder

was placed at the upstream heating zone. After evacuating the furnace to base pressure, a 10% H_2/Ar mixture was introduced as the carrier gas. Subsequently, the temperatures for MoO_2/substrate and Se were increased to 880 ℃ and 280 ℃, respectively. MoS_2 growth was proceeding at 880 ℃ for 10 minutes at 20 Torr. After growth the system was cooled down naturally to room temperature.

After CVD growth, the samples underwent ambient exposure for hours during initial inspections (e.g., optical microscopy and Raman spectroscopy) before being stored in an evacuated low-vacuum glass bulb (~10^{-2} Torr). Prior to further characterization (e.g., STM, ARPES, and STEM), the samples were retrieved from the glass bulb, resulting in an additional ambient exposure ranging from several minutes to a few hours depending on facility locations.

Characterization

STM measurements. The STM experiments were carried out in an UHV low-temperature STM system (Omicron) at a base pressure of 8×10^{-11} Torr. The STM images were acquired at 77 K in the constant-current mode by using a tungsten tip prepared by electrochemical etching in a NaOH solution. STS was carried out using a lock-in amplifier at 451.7 Hz with a 0.02 V modulation. The tip condition was checked before each set of experiments by test measurements of the surface reconstruction and surface states of an Au(111) sample to ensure consistency. The $MoSe_2$ sample was annealed at 180 ℃ in the UHV chamber to remove the Se capping for STM/S measurements. All STS and FER spectra reported in the main text were acquired on atomically clean, defect-free regions, with the tip positioned away from those detectable point defects.

ARPES Characterization. The ARPES experiments were conducted at beamline 21B1 of the Taiwan Light Source (TLS) and beamline 39A1 of the Taiwan Photon Source (TPS) in the National Synchrotron Radiation Research Center (NSRRC). $MoSe_2$/Au(111) samples with a Se capping layer were annealed in UHV at 160 °C for one hour to remove the capping layer before ARPES measurements. The ARPES data were collected in an UHV chamber equipped with a hemispherical analyzer (Scienta R4000 at TLS 21B1 and DA30L at TPS 39A1), each featuring a collection angle of ±15°. All spectra were recorded at 80 K and a base pressure of $< 1\times10^{-10}$ Torr, using an incident photon energy of 50 eV. The angular resolution was approximately 0.2°, and the overall energy resolution was better than 10 meV. Photon energy calibration was conducted by referencing the Au Fermi level.

Computational details. The first-principles density functional theory (DFT) calculations were

performed using the plane wave basis set and projector augmented-wave (PAW) potentials,[3] as implemented in the Vienna *ab initio* simulation package (VASP).[4,5] The exchange-correlation functional was treated by the generalized gradient approximation in the Perdew–Burke–Ernzerhof (PBE) form Perdew et. al.[6] Additionally, the long-range van der Waals corrections were incorporated with the DFT-D3 method of Grimme et. al.[7] The lattice constants of $MoSe_2$ and Au were taken from the experimental values and the 7×7 $MoSe_2$ on top of 8×8 Au(111) supercell geometry was simulated by a repeating slab of five Au(111) layers and a vacuum region of ~ 14.5 Å. The interface structures then lead to a lattice mismatch of 0.24%. Au atoms in the bottom two layers were fixed at bulk positions, while the top three layers and $MoSe_2$ were allowed full relaxation. The kinetic energy cutoff was set at 400 eV and a Monkhorst-Pack 6×6×1 k-point mesh was used to sample the surface Brillouin zone. The unfolding band structures were calculated using the band-unfolding method as implemented in the BandUP code.[8,9]

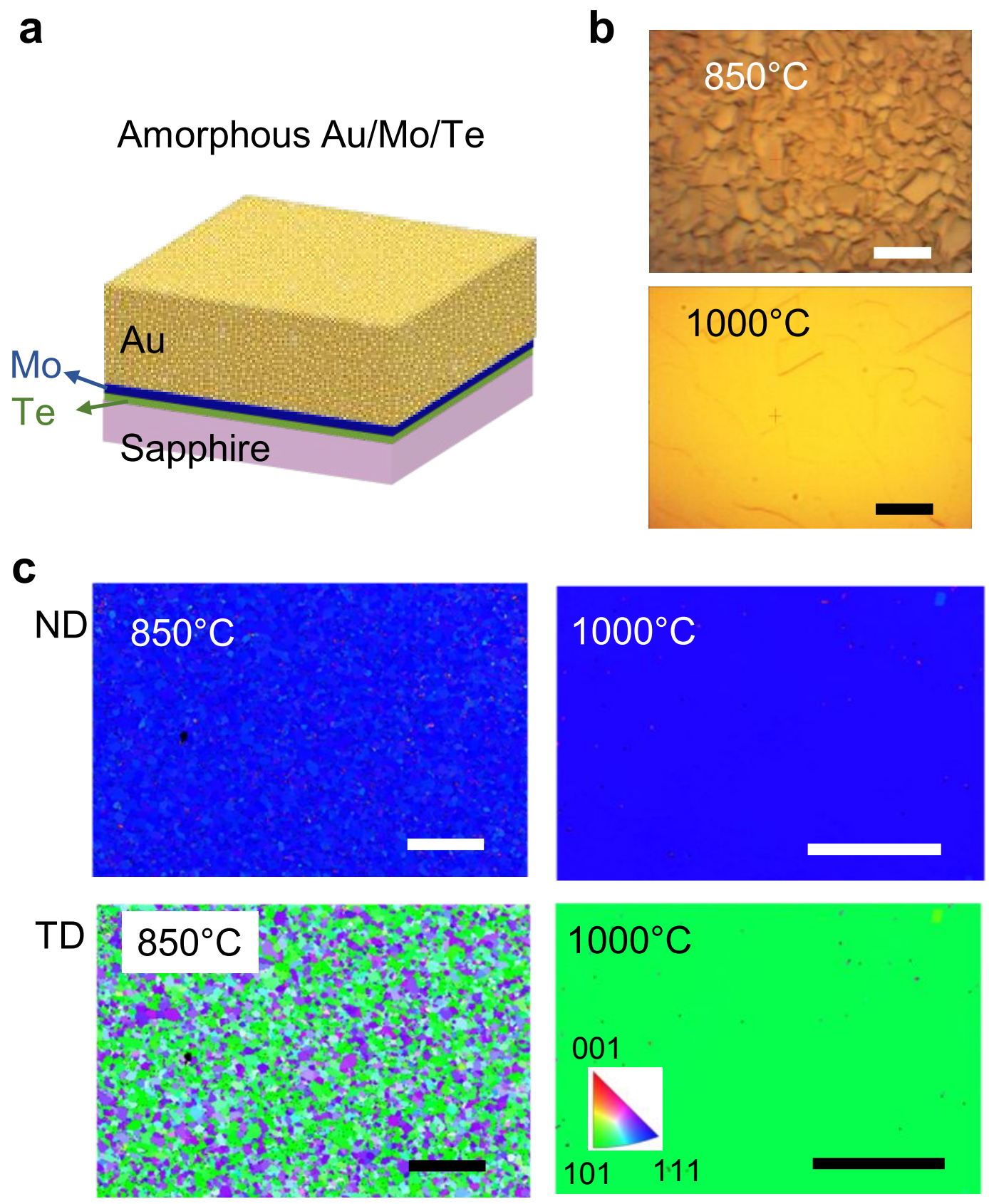

Figure S1. The formation of Au(111) surface. a Schematic of the substrate structure. **b** Optical microscope images of the Au surface after annealing at 850 °C and 1,000 °C. The scale bars are 10 µm. **c** EBSD inverse pole figure maps in the normal direction and transverse direction for Au surface after annealing at 850 °C and 1,000 °C. The scale bar is 100 µm for 850°C and 250 µm for 1,000 °C.

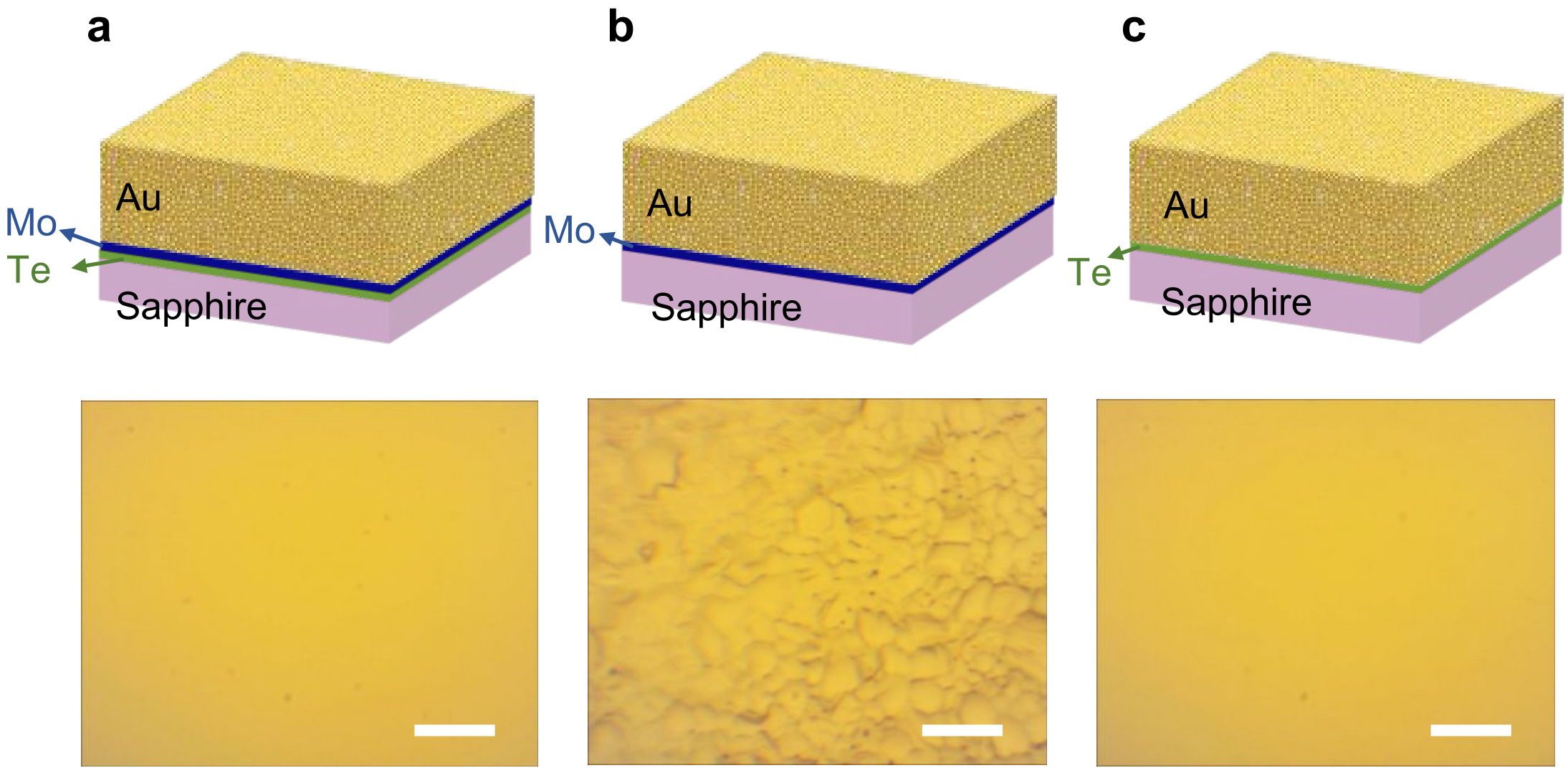

Figure S2. Preparation of Au and Mo-Au alloy substrates. Optical microscope images of **a** Au/Te/Sapphire, **b** Au/Mo/Te/Sapphire, **c** Au/Mo/Sapphire samples after annealing at 1,000 °C. The scale bars are 10 μm.

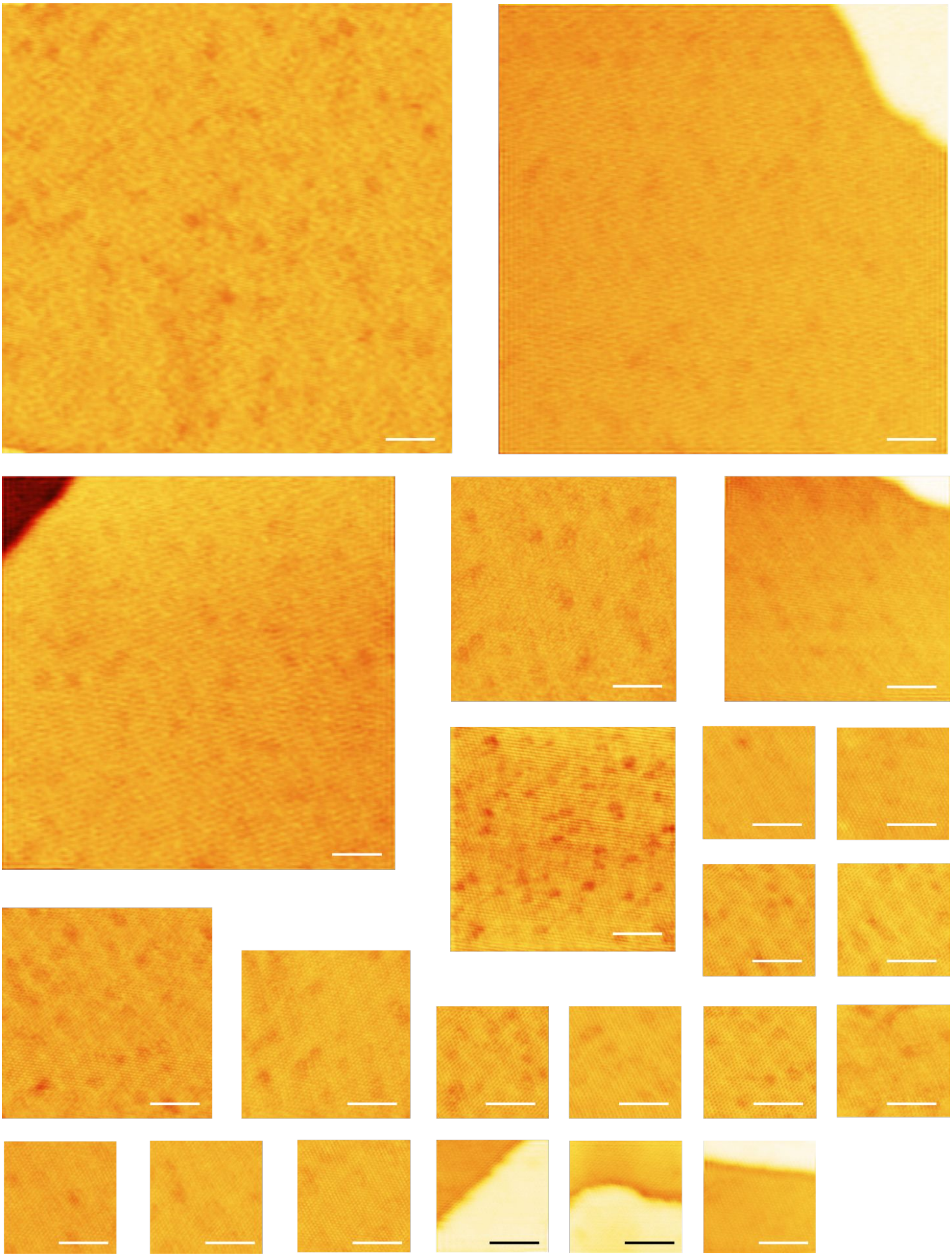

Figure S3. The STM images acquired from ultralow defect density $MoSe_2$/Au(111). The scale bars are 4 nm. The scan bias for each is −5 mV.

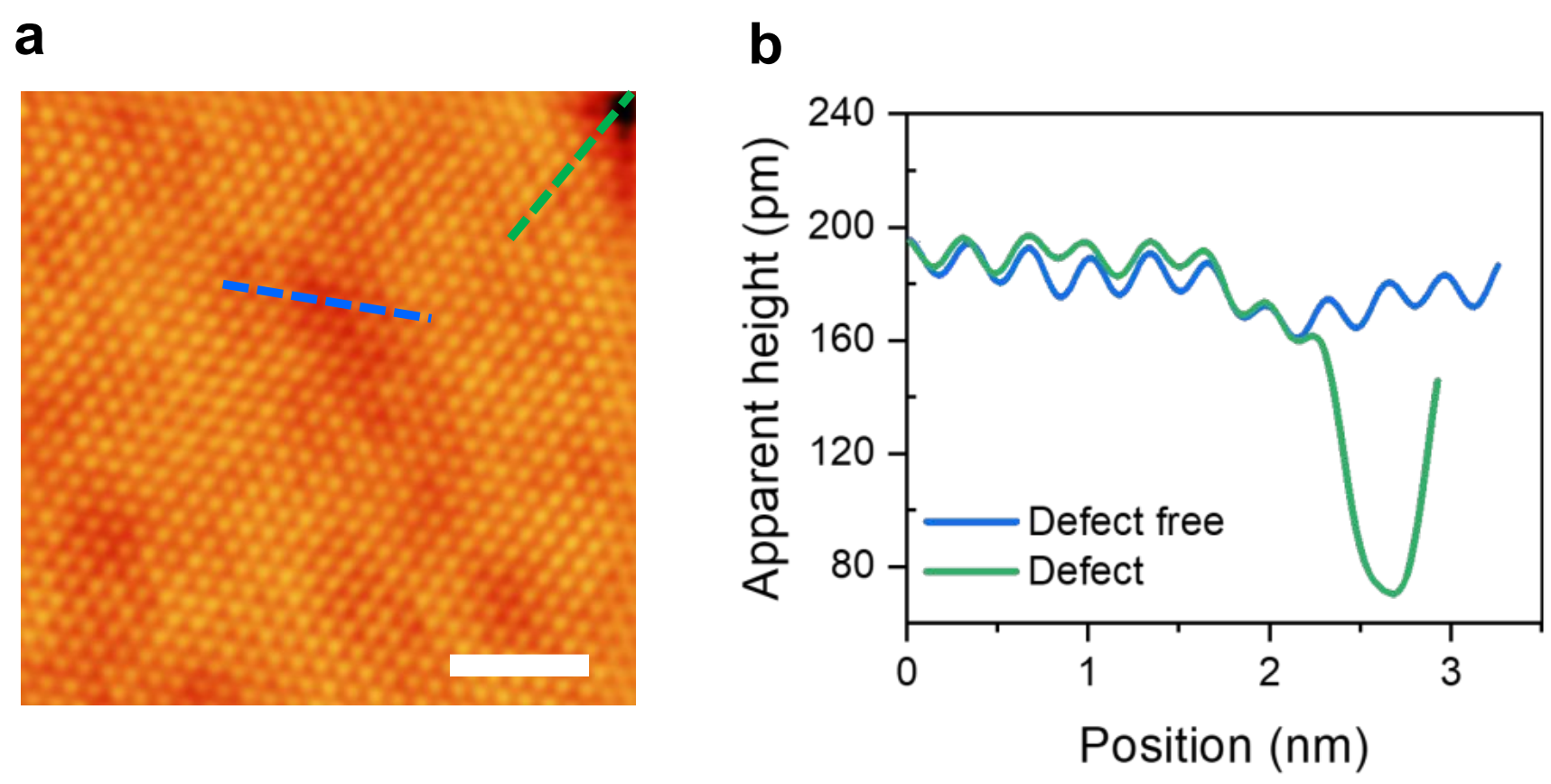

Figure S4. The defect characterization in $MoSe_2$ with an ultralow defect density.
a The STM image of defect observed in $MoSe_2$ grown by oxygen-free approach and Se-rich cooling process. The scale bar is 2 nm. The scan bias is −5 mV. **b** The corresponding line scan profile of the defect and defect-free region.

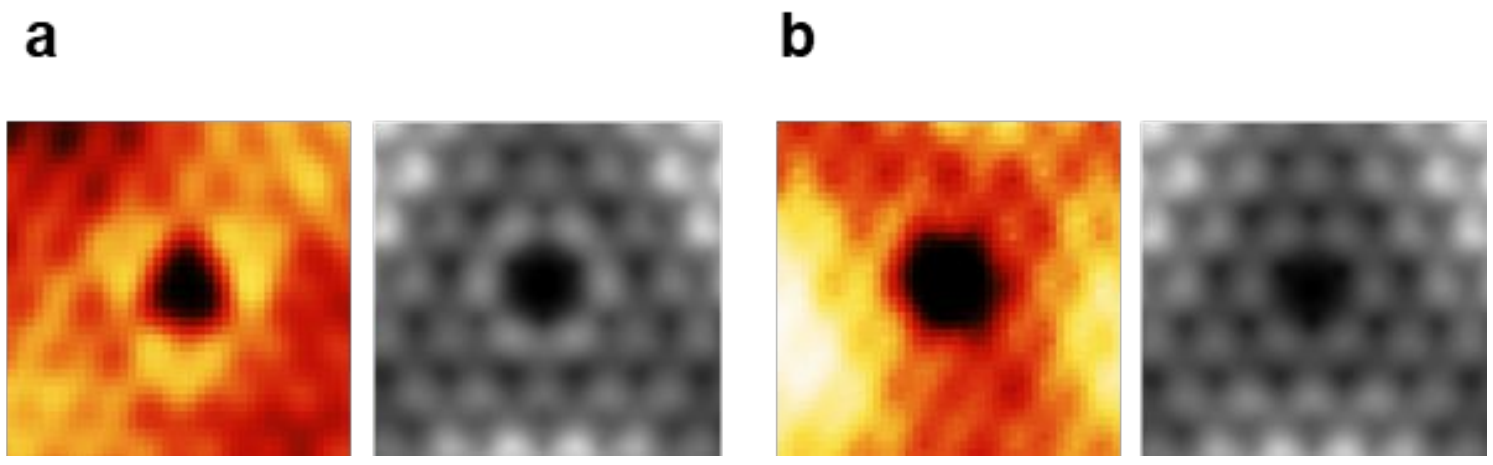

Figure S5. Experimental and simulated STM images of defects.
Comparisons of STM image (left) and DTF simulated image (right) of **a** Se vacancy; **b** oxygen substitution. The bias of STM image is −1.3V.

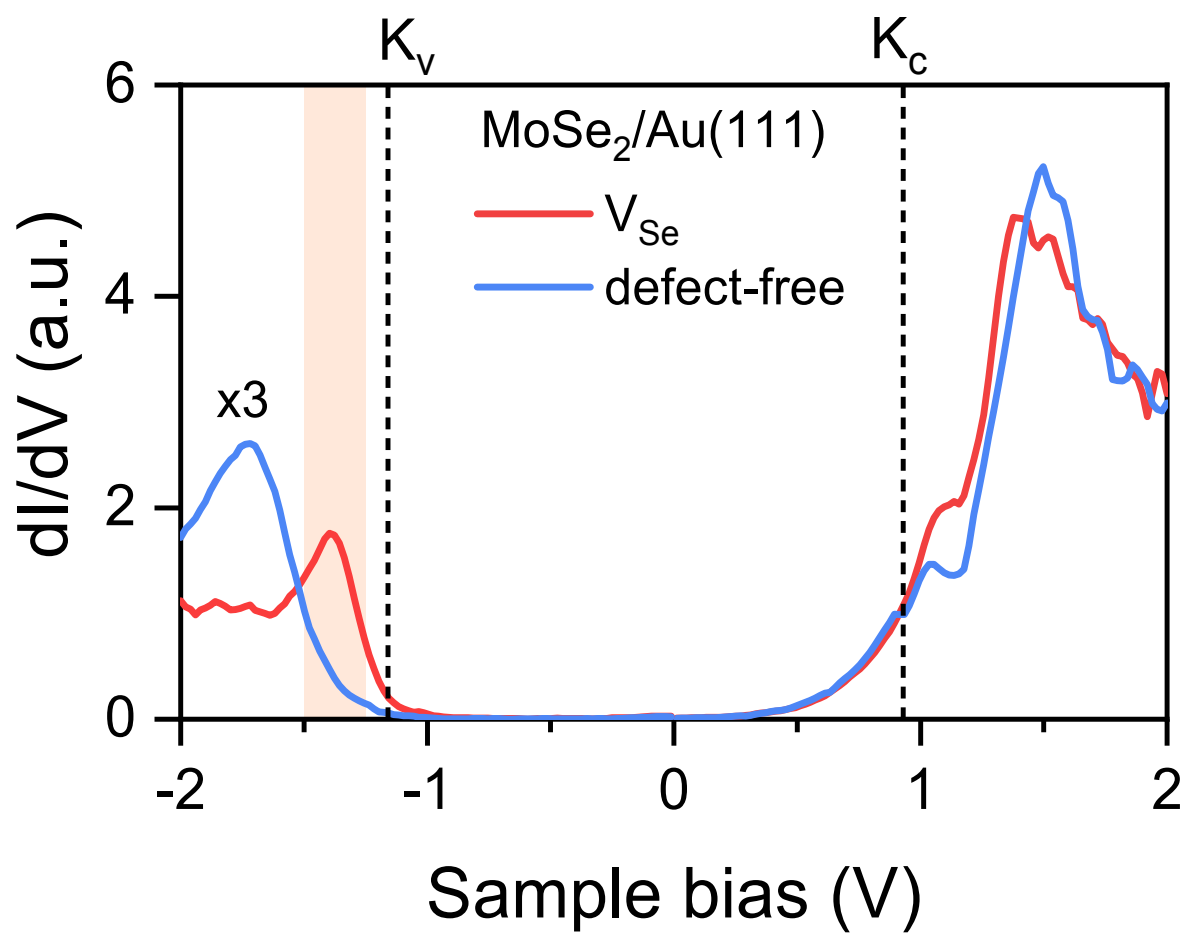

Figure S6. STS spectra acquired on an isolated V_{Se} and on defect-free regions in the deintercalated $MoSe_2$/Au(111) sample.

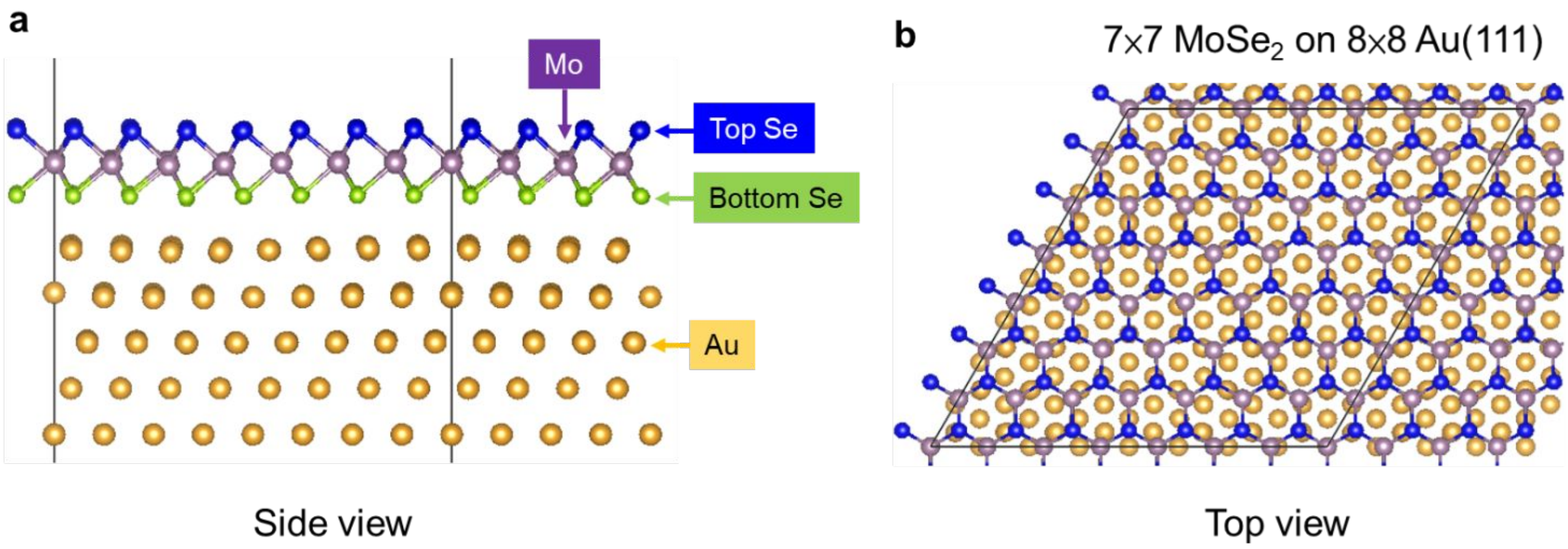

Figure S7. The calculation model for $MoSe_2$ on Au(111) supercell.
a Side view of monolayer $MoSe_2$ on five-layer Au. **b** Top view of (a).

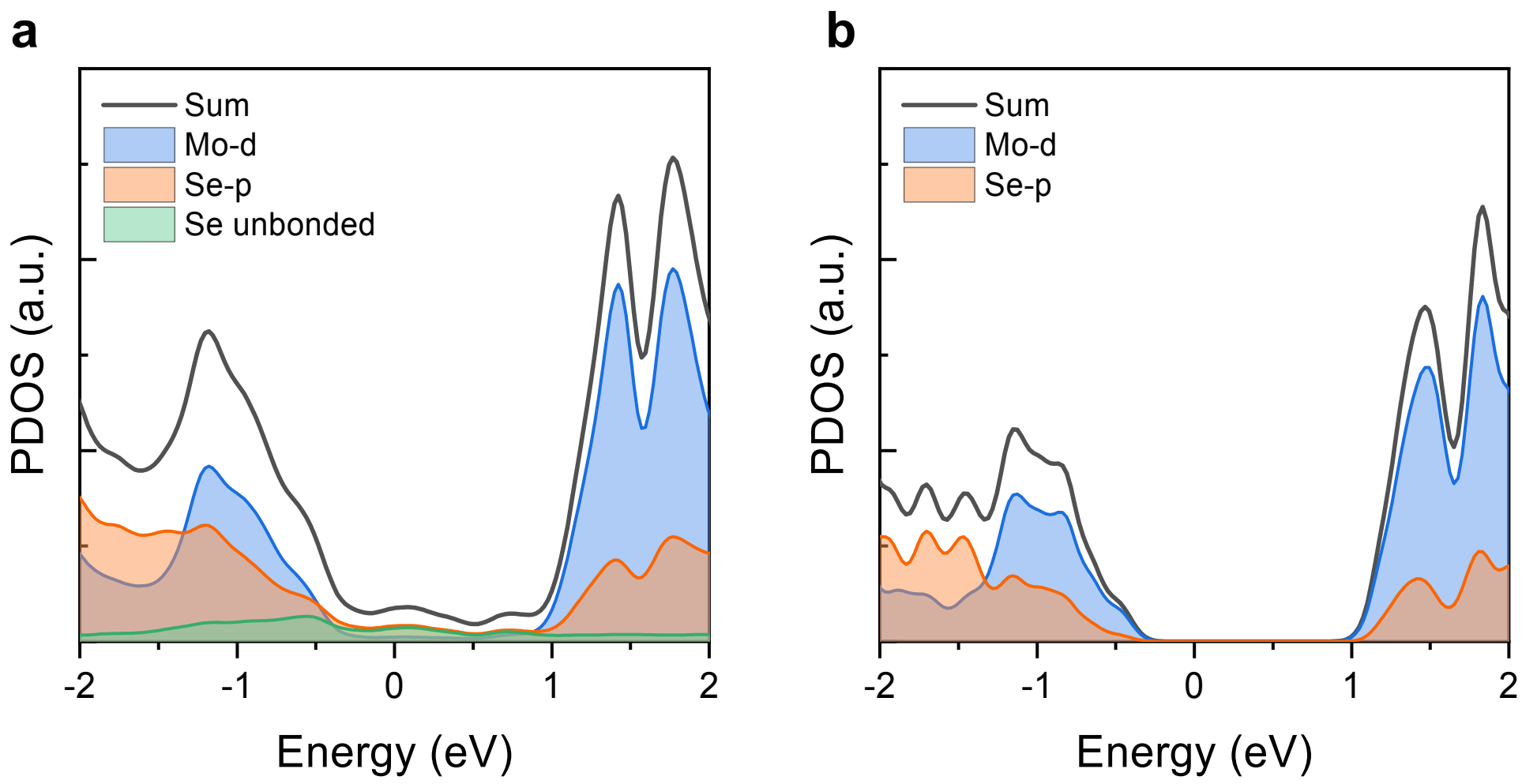

Figure S8. The calculated PDOS. a $MoSe_2$/Se/Au(111). **b** Free standing of $MoSe_2$.

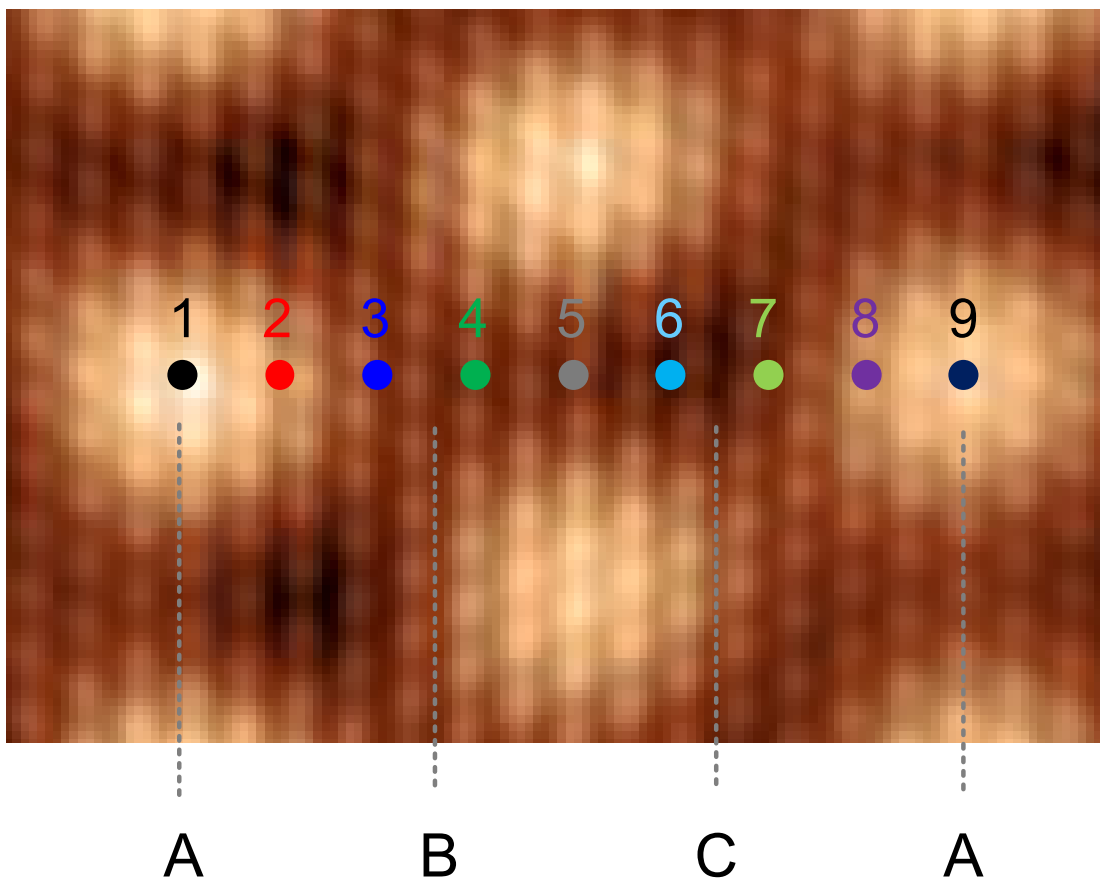

Figure S9 The atomically-resolved STM image of moiré supercell. The labeled positions 1-9 correspond to the measurement positions in Fig. 5c. The scan bias is –5 mV.

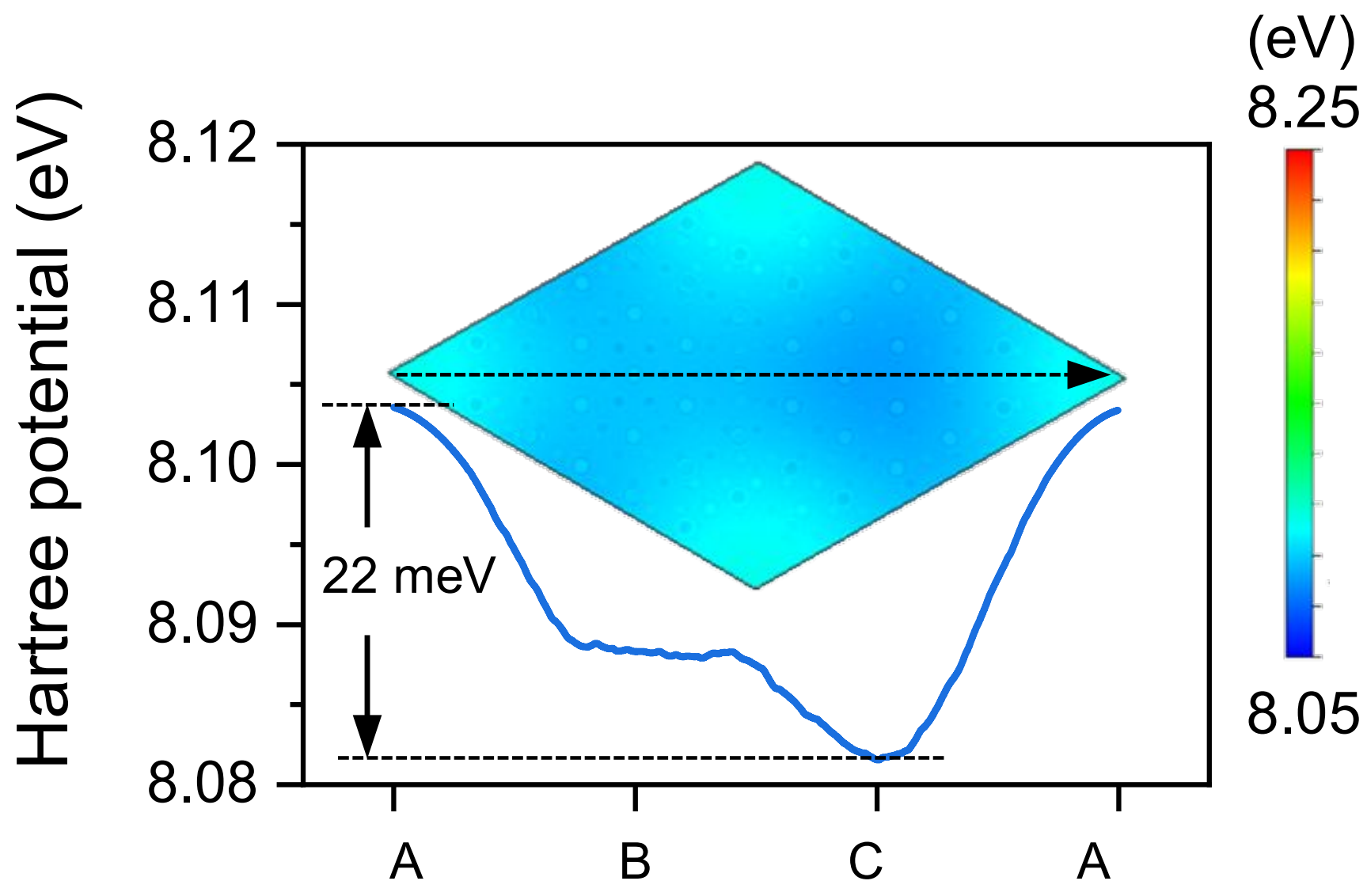

Figure S10 The calculated Hartree potential across the moiré supercell. The inset shows the contour plot of the Hartree potential in a supercell. The largest potential variation is 22 meV.

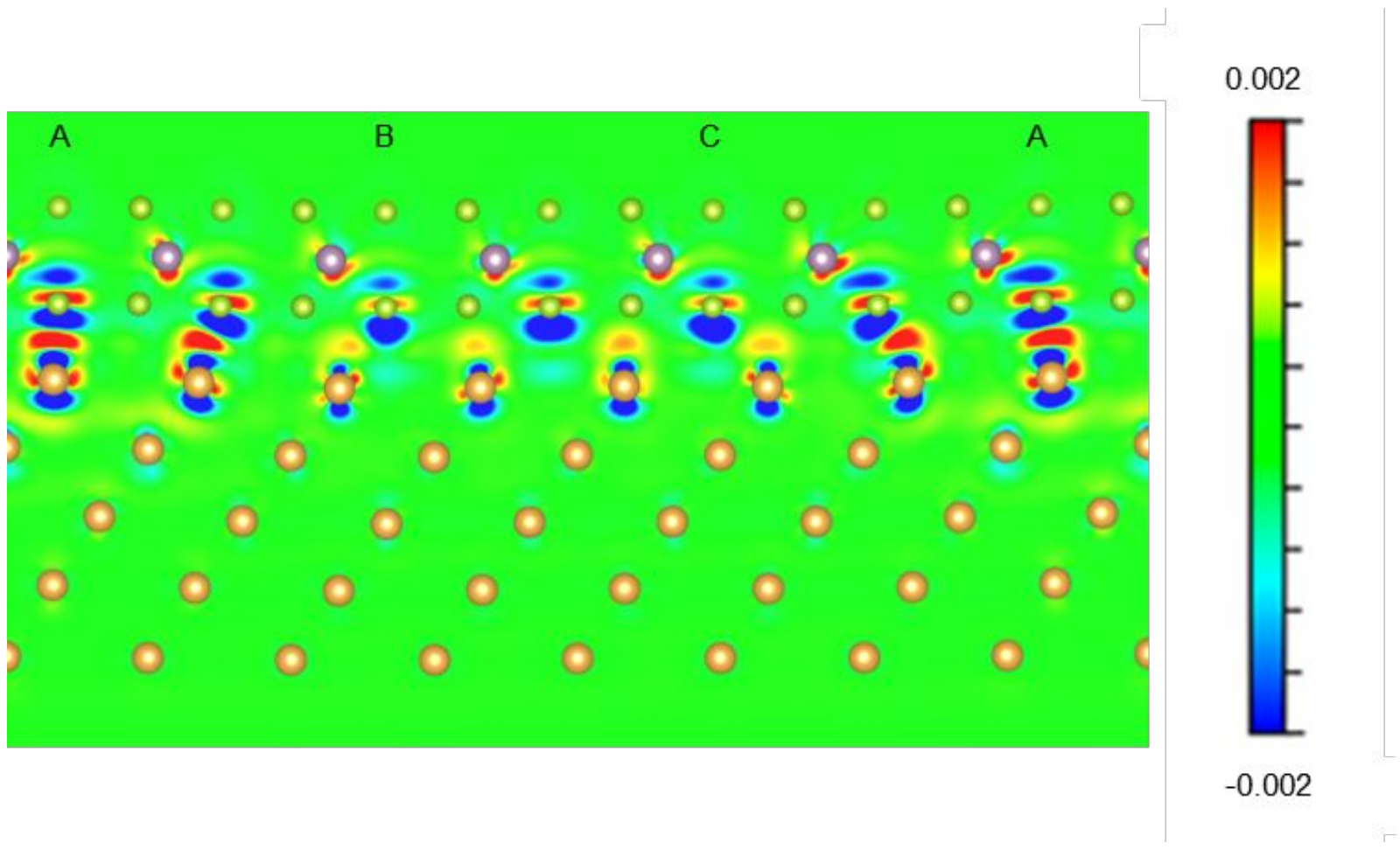

Figure S11. The charge density difference in a moiré supercell of $MoSe_2$/Au(111).

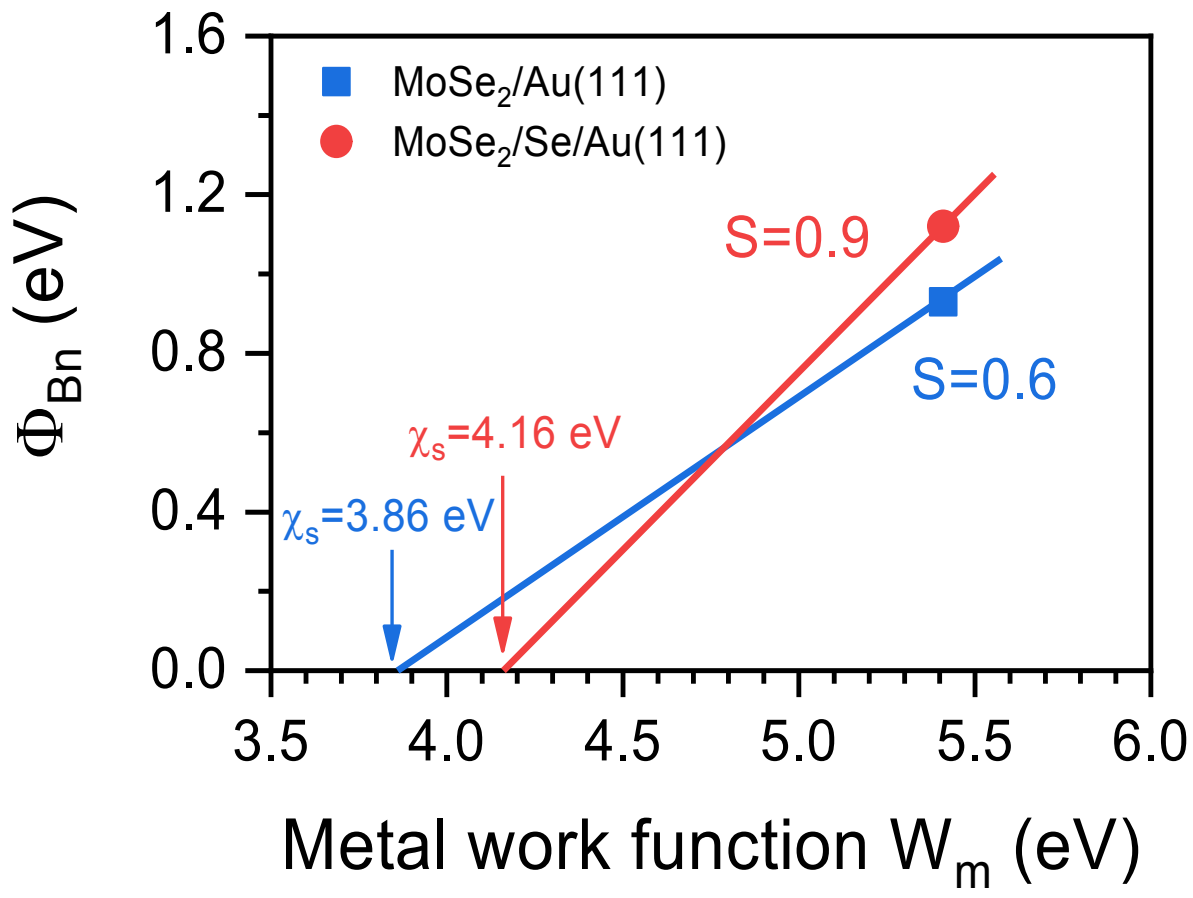

Figure S12. Estimation of the pinning factor S for $MoSe_2$/Au(111) interface with and without Se intercalation. The estimation is based on the measured SBH Φ_{Bn} for Au (W_m $= 5.41$ eV) and considering $\Phi_{Bn} = 0$ at $W_m = \chi_s$. See Methods in the main text for the procedure and its limitations.

REFERENCES

1. Okamoto, H.; Massalski, T. B. The Au–Te (Gold–Tellurium) System. *Bull. Alloy Phase Diagr.* **1984**, *5* (2), 172–177.
2. Shivayogimath, A.; Thomsen, J. D.; Mackenzie, D. M. A.; Geisler, M.; Stan, R.-M.; Holt, A. J.; Bianchi, M.; Crovetto, A.; Whelan, P. R.; Carvalho, A.; Neto, A. H. C.; Hofmann, P.; Stenger, N.; Bøggild, P.; Booth, T. J. A Universal Approach for the Synthesis of Two-Dimensional Binary Compounds. *Nat. Commun.* **2019**, *10* (1), 2957.
3. Kresse, G.; Joubert, D. From Ultrasoft Pseudopotentials to the Projector Augmented-Wave Method. *Phys. Rev. B* **1999**, *59* (3), 1758–1775.
4. Kresse, G.; Hafner, J. Ab initio molecular dynamics for liquid metals. *Phys. Rev. B Condens. Matter* **1993**, 47, 558–561.
5. Kresse, G.; Furthmüller, J. Efficiency of ab-initio total energy calculations for metals and semiconductors using a plane-wave basis set. *Comput. Mater. Sci.* **1996**, *6*, 15–50.
6. Perdew, J. P.; Burke, K.; Ernzerhof, M. Generalized gradient approximation made simple. *Phys. Rev. Lett.* **1996**, *77*, 3865–3868.
7. Grimme, S.; Antony, J.; Ehrlich, S.; Krieg, H. A consistent and accurate ab initio parametrization of density functional dispersion correction (DFT-D) for the 94 elements H-Pu. *J. Chem. Phys.* **2010**, *132*, 154104.
8. Medeiros, P. V. C.; Stafström, S.; Björk, J. Effects of extrinsic and intrinsic perturbations on the electronic structure of graphene: Retaining an effective primitive cell band structure by band unfolding. *Phys. Rev. B Condens. Matter* **2014**, *89*, 041407.
9. Medeiros, P. V. C.; Tsirkin, S. S.; Stafström, S.; Björk, J. Unfolding spinor wave functions and expectation values of general operators: Introducing the unfolding-density operator. *Phys. Rev. B Condens. Matter* **2015**, *91*, 041116.
